# BRSK2 in pancreatic **β** cells promotes hyperinsulinemia-coupled insulin resistance and its genetic variants are associated with human type 2 diabetes

**DOI:** 10.1093/jmcb/mjad033

**Published:** 2023-05-15

**Authors:** Rufeng Xu, Kaiyuan Wang, Zhengjian Yao, Yan Zhang, Li Jin, Jing Pang, Yuncai Zhou, Kai Wang, Dechen Liu, Yaqin Zhang, Peng Sun, Fuqiang Wang, Xiaoai Chang, Tengli Liu, Shusen Wang, Yalin Zhang, Shuyong Lin, Cheng Hu, Yunxia Zhu, Xiao Han

**Affiliations:** Key Laboratory of Human Functional Genomics of Jiangsu Province, Nanjing Medical University, Nanjing 211166, China; Key Laboratory of Human Functional Genomics of Jiangsu Province, Nanjing Medical University, Nanjing 211166, China; Key Laboratory of Human Functional Genomics of Jiangsu Province, Nanjing Medical University, Nanjing 211166, China; Key Laboratory of Human Functional Genomics of Jiangsu Province, Nanjing Medical University, Nanjing 211166, China; Institute for Metabolic Disease, Fengxian Central Hospital Affiliated to Southern Medical University, Shanghai 201499, China; Shanghai Diabetes Institute, Shanghai Key Laboratory of Diabetes Mellitus, Shanghai Clinical Center for Diabetes, Shanghai Sixth People's Hospital Affiliated to Shanghai Jiao Tong University School of Medicine, Shanghai 200233, China; Key Laboratory of Human Functional Genomics of Jiangsu Province, Nanjing Medical University, Nanjing 211166, China; Key Laboratory of Human Functional Genomics of Jiangsu Province, Nanjing Medical University, Nanjing 211166, China; Key Laboratory of Human Functional Genomics of Jiangsu Province, Nanjing Medical University, Nanjing 211166, China; Key Laboratory of Human Functional Genomics of Jiangsu Province, Nanjing Medical University, Nanjing 211166, China; Key Laboratory of Human Functional Genomics of Jiangsu Province, Nanjing Medical University, Nanjing 211166, China; Key Laboratory of Human Functional Genomics of Jiangsu Province, Nanjing Medical University, Nanjing 211166, China; Analysis Center, Nanjing Medical University, Nanjing 210029, China; Key Laboratory of Human Functional Genomics of Jiangsu Province, Nanjing Medical University, Nanjing 211166, China; Organ Transplant Center, Tianjin First Central Hospital, Nankai University, Tianjin 300192, China; Organ Transplant Center, Tianjin First Central Hospital, Nankai University, Tianjin 300192, China; State Key Laboratory for Cellular Stress Biology, School of Life Sciences, Xiamen University, Xiamen 361102, China; State Key Laboratory for Cellular Stress Biology, School of Life Sciences, Xiamen University, Xiamen 361102, China; Institute for Metabolic Disease, Fengxian Central Hospital Affiliated to Southern Medical University, Shanghai 201499, China; Shanghai Diabetes Institute, Shanghai Key Laboratory of Diabetes Mellitus, Shanghai Clinical Center for Diabetes, Shanghai Sixth People's Hospital Affiliated to Shanghai Jiao Tong University School of Medicine, Shanghai 200233, China; Key Laboratory of Human Functional Genomics of Jiangsu Province, Nanjing Medical University, Nanjing 211166, China; Key Laboratory of Human Functional Genomics of Jiangsu Province, Nanjing Medical University, Nanjing 211166, China

**Keywords:** type 2 diabetes mellitus, genetic variant, BRSK2, β-cell hypersecretion, hyperinsulinemia, insulin resistance

## Abstract

Brain-specific serine/threonine-protein kinase 2 (BRSK2) plays critical roles in insulin secretion and β-cell biology. However, whether BRSK2 is associated with human type 2 diabetes mellitus (T2DM) has not been determined. Here, we report that BRSK2 genetic variants are closely related to worsening glucose metabolism due to hyperinsulinemia and insulin resistance in the Chinese population. BRSK2 protein levels are significantly elevated in β cells from T2DM patients and high-fat diet (HFD)-fed mice due to enhanced protein stability. Mice with inducible β-cell-specific *Brsk2* knockout (βKO) exhibit normal metabolism with a high potential for insulin secretion under chow-diet conditions. Moreover, βKO mice are protected from HFD-induced hyperinsulinemia, obesity, insulin resistance, and glucose intolerance. Conversely, gain-of-function BRSK2 in mature β cells reversibly triggers hyperglycemia due to β-cell hypersecretion-coupled insulin resistance. Mechanistically, BRSK2 senses lipid signals and induces basal insulin secretion in a kinase-dependent manner. The enhanced basal insulin secretion drives insulin resistance and β-cell exhaustion and thus the onset of T2DM in mice fed an HFD or with gain-of-function BRSK2 in β cells. These findings reveal that BRSK2 links hyperinsulinemia to systematic insulin resistance via interplay between β cells and insulin-sensitive tissues in the populations carrying human genetic variants or under nutrient-overload conditions.

## Introduction

Type 2 diabetes mellitus (T2DM) is a worldwide epidemic. Its key features are hyperinsulinemia and hyperglycemia due to insulin resistance and pancreatic β-cell dysfunction (Kahn et al., 2006). In general, glycemic homeostasis is achieved by a balance between glucose production in the liver and peripheral glucose metabolism. This balance depends on a series of processes regulated by insulin and glucagon secretion from islet β and α cells, coupled with feeding and fasting (Gromada et al., 2018). Hormonal inability to suppress hepatic glucose production (HGP) or to store energy postprandially as fat in adipose tissues leads to hyperglycemia (Roden and Shulman, 2019). During T2DM progression, obesity and insulin resistance increase insulin secretion to trigger a vicious cycle of hyperinsulinemia and insulin resistance that ultimately results in β-cell failure via β-cell degranulation, trans/dedifferentiation, and, to some extent, apoptosis (Remedi and Emfinger, 2016). The cause-and-effect relationship between hyperinsulinemia and insulin resistance remains unresolved and hotly debated (Page and Johnson, 2018). Identification of factors that trigger the development of hyperinsulinemia independent of insulin resistance may help to unravel this dilemma and further clarify the mechanism of T2DM.

Hyperinsulinemia is maintained for many years to compensate for insulin resistance prior to the onset of frank diabetes (Shanik et al., 2008). β cells are equipped with glucose-sensing machinery, which allows these cells to sense blood glucose levels, secrete an appropriate amount of insulin, and synthesize the insulin protein needed to refill secretory granules (Boland et al., 2017). A deficiency in glucose-stimulated insulin secretion (GSIS) is an early manifestation of T2DM; consequently, hyperglycemia is not solely responsible for β-cell exhaustion. Other nutritional and hormonal factors may also participate in β-cell failure through secretion-uncoupled insulin biosynthesis. Notably, most of these insulinotropic factors, including free fatty acids (FFAs), glucagon-like peptide 1 (GLP-1), and gastric inhibitory polypeptide (GIP), promote insulin secretion by activating different G-protein-coupled receptors (GPCRs) (Moran et al., 2016; Sabrautzki et al., 2017; Jones et al., 2018). Ligand-activated GPCRs enhance insulin secretion through mechanisms that are either dependent or independent of the blood glucose level (Oh and Olefsky, 2016), thus leading to condescending hyperinsulinemia and β-cell failure. However, molecules that transduce insulinotropic signaling are still unclear.

Brain-specific serine/threonine-protein kinase 2 (BRSK2) is a member of the adenosine monophosphate-activated protein kinase (AMPK)-related kinase family. Selectively expressed in the pancreas and brain (Nie et al., 2013b), BRSK2 and its highly conserved BRSK1 isoform define neuronal polarization and form central axon arbors for sensory neurons (Kishi et al., 2005; Lilley et al., 2013). BRSK2 also plays key roles in sensing nutrient signals and may lead to obesity and metabolic disorders (Nie et al., 2013a; Tamir et al., 2020). Mice with whole-body knockout of *Brsk2* remain healthy and fertile but exhibit growth retardation and hypoinsulinemia. Mice with pancreas-specific *Brsk2* deletion exhibit oral glucose intolerance but maintain normal glucose excursion after intraperitoneal (*i.p.*) glucose injection due to loss of the gut hormone GLP-1 (Nie et al., 2013b). As a mediator of mTORC1 signaling, either global or pancreatic *Brsk2* deletion reduces islet mass and β-cell size (Nie et al., 2012, 2013c, 2018). At present, the role of BRSK2 in mature β cells and the pathogenesis of T2DM remains poorly understood.

The aim of the present study was to assess the *in vivo* role of BRSK2 in β-cell function and the development of T2DM. We used inducible gain-of-function and loss-of-function variants of β-cell *Brsk2* to show that the amount of BRSK2 in mature β cells was positively associated with hyperinsulinemia, insulin resistance, and the onset of T2DM. Notably, human genetic analysis identifies at least three BRSK2 variants closely associated with worsening glucose metabolism, indicating a fundamental role of BRSK2 in human T2DM.

## Results

### Human BRSK2 locus variants are associated with worsening glucose metabolism in the Chinese population

**Figure 1:**
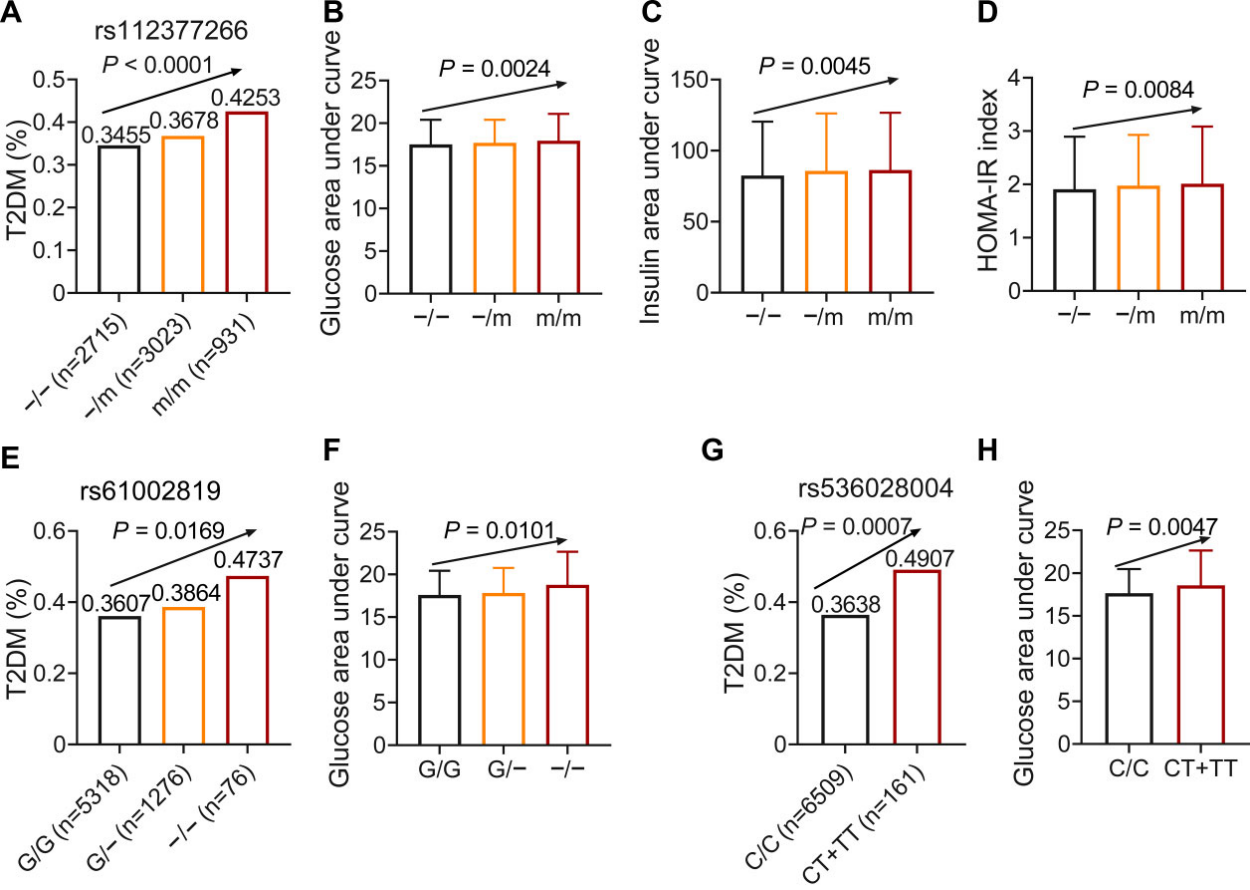
Human *BRSK2* loci variants are associated with worsening glucose metabolism. (**A**–**D**) The incidence of T2DM (**A**), plasma glucose level after OGTT, demonstrated by the area under curve (AUC) (**B**), plasma insulin level after OGTT (**C**), and HOMA-IR index (**D**) in rs112377266 variant carriers. (**E** and **F**) The incidence of T2DM (**E**) and plasma glucose level after OGTT (**F**) in rs61002819 variant carriers. (**G** and **H**) The incidence of T2DM (**G**) and plasma glucose level after OGTT (**H**) in rs536028004 variant carriers.

We first analyzed the metabolic traits in Shanghai Nicheng Cohort Study (Supplementary Table S1). After screening genetic variants of the human gene *BRSK2*, we identified three variants associated with diabetes mellitus and related glucose metabolic traits, including rs112377266 (Figure 1A–D, −>dup(G)_4_CTCACCTGTGG, an insertion variant in intron 15 of the *BRSK2* gene, minor allele frequency = 0.37), rs61002819 (Figure 1E and F, G>−, a deletion variant in the splice region, variant frequency = 0.11), and rs536028004 (Figure 1G and H, C>T, a 3′-untranslated region (3′-UTR) variant, variant frequency = 0.01). The linkage disequilibrium relationships are shown in Supplementary Table S2.

We analyzed the plasma glucose and insulin levels during 75 g oral glucose tolerance tests (OGTTs) at 0, 30, and 120 min. Insulin sensitivity and β-cell function were evaluated by using serum insulin and blood glucose data. We included two methods, the homeostatic model of insulin resistance (HOMA-IR) and the Gutt insulin sensitivity index (Gutt-ISI), to evaluate the fasting and post-glucose loading insulin sensitivities of these human subjects. We used Stumvoll 2^nd^ insulin secretion to evaluate β-cell function.

Notably, subjects with minor alleles had a higher risk of T2DM, indicating the important role of *BRSK2* in glucose metabolic process. Specifically, rs112377266 carriers had a higher incidence of T2DM (Figure 1A, −/− homozygote: 34.55%; −/dup(G)_4_CTCACCTGTGG heterozygote: 36.78%; dup(G)_4_CTCACCTGTGG homozygote: 42.53%). The results also showed that dup(G)_4_CTCACCTGTGG carriers had higher plasma glucose and plasma insulin levels (Figure 1B and C; Supplementary Figure S1A and B), indicating the involvement of insulin resistance in the onset of T2DM. Logistic regression and multiple linear regression showed that rs112377266 was associated with T2DM (odds ratio (OR) and 95% confidence interval (CI) = 1.04 [1.02–1.05], *P *= 2.42 × 10^−5^) after adjusting for age and sex. The HOMA-IR index results revealed that dup(G)_4_CTCACCTGTGG carriers exhibited insulin resistance (Figure 1D), while the Gutt-ISI results were the same for all subjects (Supplementary Figure S1C). However, dup(G)4CTCACCTGTGG carriers showed diminished Stumvoll 2^nd^ insulin secretion (β = −3.19, SE = 0.97, *P *= 9.87 × 10^−4^; Figure 1C; Supplementary Figure S1B and D). Therefore, this variant may drive T2DM through hyperinsulinemia, β-cell dysfunction, and insulin resistance.

Another splice-region variant rs61002819 was also associated with T2DM (OR and 95% CI = 1.13 [1.04–1.05], *P *= 9.84 × 10^−4^). Subjects with a G deletion had a higher incidence of T2DM (G/G homozygote: 36.07%; G/− heterozygote: 38.64%; −/− homozygote: 47.37%). OGTT results also indicated impaired glucose excursion in rs61002819 variant carriers (Figure 1F; Supplementary Figure S1E). The plasma insulin levels determined during OGTTs and the calculated Stumvoll 2^nd^ insulin secretion were not significantly altered (Supplementary Figure S1F and G). The Gutt-ISI results showed insulin insensitivity in G>− deletion carriers (Supplementary Figure S1H). Thus, the rs61002819 variant is associated with insulin resistance.

Furthermore, the rs536028004 variant located in 3′-UTR had a relatively low frequency. The T allele carriers had a higher incidence of T2DM (Figure 1G, CC homozygote: 36.38%; T allele carriers: 49.07%), glucose intolerance (Figure 1H; Supplementary Figure S1I), and insulin resistance (Supplementary Figure S1L), while plasma insulin levels during OGTTs were unaffected (Supplementary Figure S1J and K). After adjustment for age and sex, rs536028004 was also associated with T2DM (OR and 95% CI = 1.03 [1.01–1.06], *P *= 9.90 × 10^−3^). This variant is also associated with T2DM due to insulin resistance.

Taken together, BRSK2 locus variants potentially play roles in regulating glucose metabolism in humans to be associated with both hyperinsulinemia and insulin resistance.

### BRSK2 protein levels are elevated in β cells from T2DM patients and diet-induced obese mice

**Figure 2:**
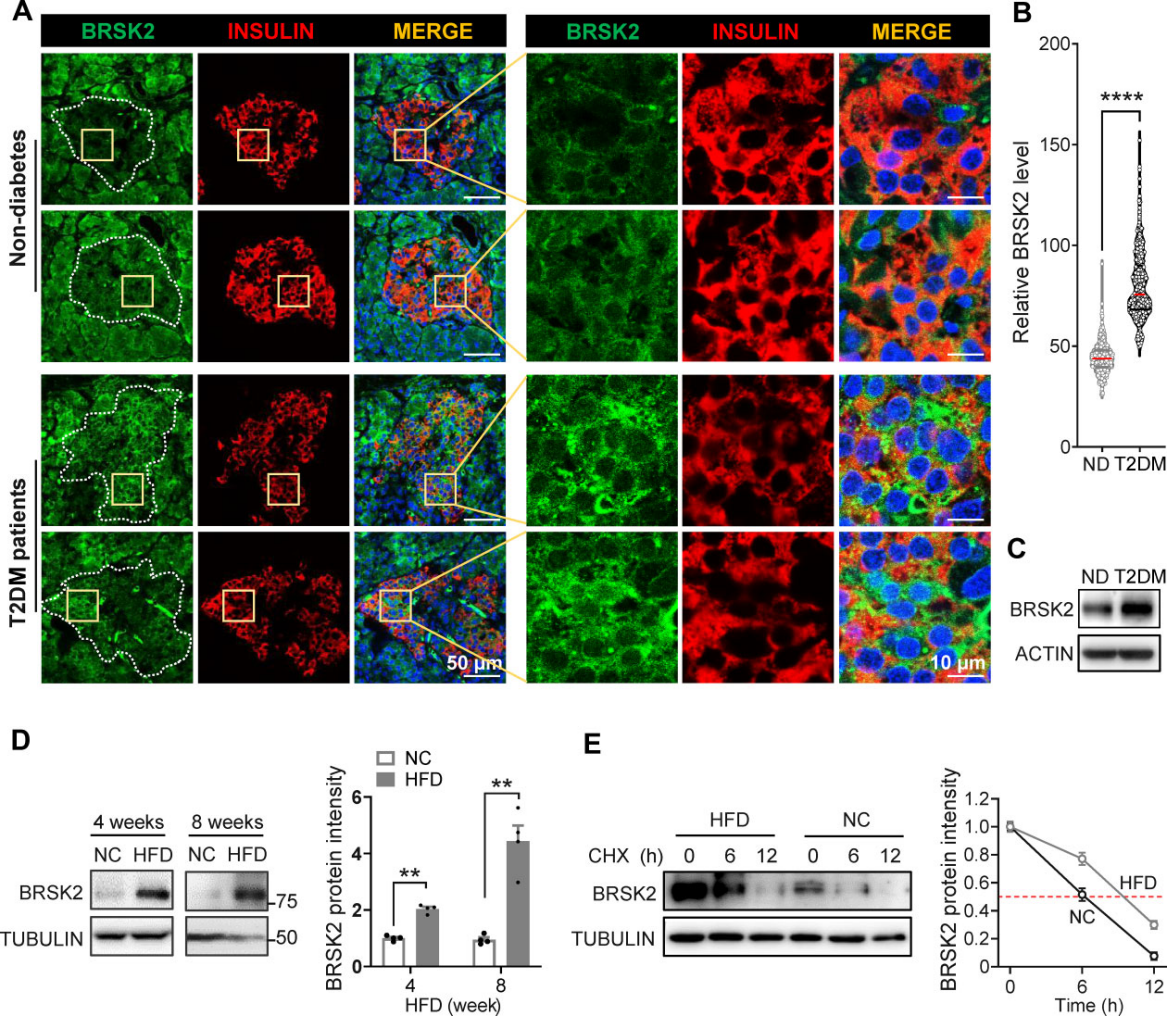
BRSK2 protein levels are elevated in β cells from T2DM patients and diet-induced obese mice. (**A**) Representative immunofluorescence images of BRSK2 in human pancreases obtained from non-diabetes (ND) donors and T2DM patients (*n* = 4 samples each). (**B**) Violin plot of green fluorescent intensities in INSULIN-positive β cells (*n* = 641 β cells for ND, *n* = 496 β cells for T2DM). (**C**) Western blot analysis of BRSK2 in human primary islets from ND donors and T2DM patients. (**D**) Western blot analysis of BRSK2 in mouse primary islets from normal chow diet (NC) and HFD groups (*n* = 3 mice per group), with quantitated relative BRSK2 protein levels shown on the right. (**E**) Western blot analysis of BRSK2 protein stability in mouse primary islets from NC and HFD groups treated with cycloheximide (CHX, 50 μM) for the indicated time, with quantitated half-lives of BRSK2 protein shown on the right. Data are presented as mean ± SEM. **P < 0.01, ****P < 0.0001.

Next, we examined the BRSK2 protein expression in human pancreas slices from T2DM patients and non-diabetes donors. We observed that the BRSK2 protein was distributed throughout the whole pancreas. Co-staining of BRSK2 with INSULIN showed their colocalization in β cells, and the amount of BRSK2 significant increased in β cells from T2DM patients (Figure 2A and B). We also confirmed a higher BRSK2 protein level in human primary islets from T2DM patients compared with that in non-diabetic subjects (Figure 2C).

The possible associations between BRSK2 and the initiation of hyperinsulinemia and insulin resistance were assessed by determining the mRNA and protein levels of BRSK2 in islets from mice fed a high-fat diet (HFD) for different durations. As expected, HFD feeding progressively increased body weight, blood glucose, and insulin levels, causing hyperglycemia and hyperinsulinemia, compared to normal chow diet feeding (Supplementary Figure S2A–C). Primary islets from mice fed an HFD for 4 and 8 weeks showed 2- and 4-fold increase in BRSK2 protein levels, respectively, compared to that in the age- and gender-matched littermates fed an chow diet (Figure 2D). The increase in the BRSK2 protein level in islets continued after 12–16 weeks of HFD feeding (Supplementary Figure S2D). In contrast, the *BRSK2* mRNA levels were not altered in primary islets from either T2DM patients or HFD-fed mice (Supplementary Figure S2E and F). Indeed, the stability of the BRSK2 protein in HFD islets clearly increased (Figure 2E). Taken together, we conclude that BRSK2 protein levels in β cells are elevated in diabetic models and patients, likely as a result of increased protein stability.

### Loss-of-function BRSK2 in β cells improves systemic insulin sensitivity in HFD-fed mice but has little effect on chow diet-fed mice

**Figure 3:**
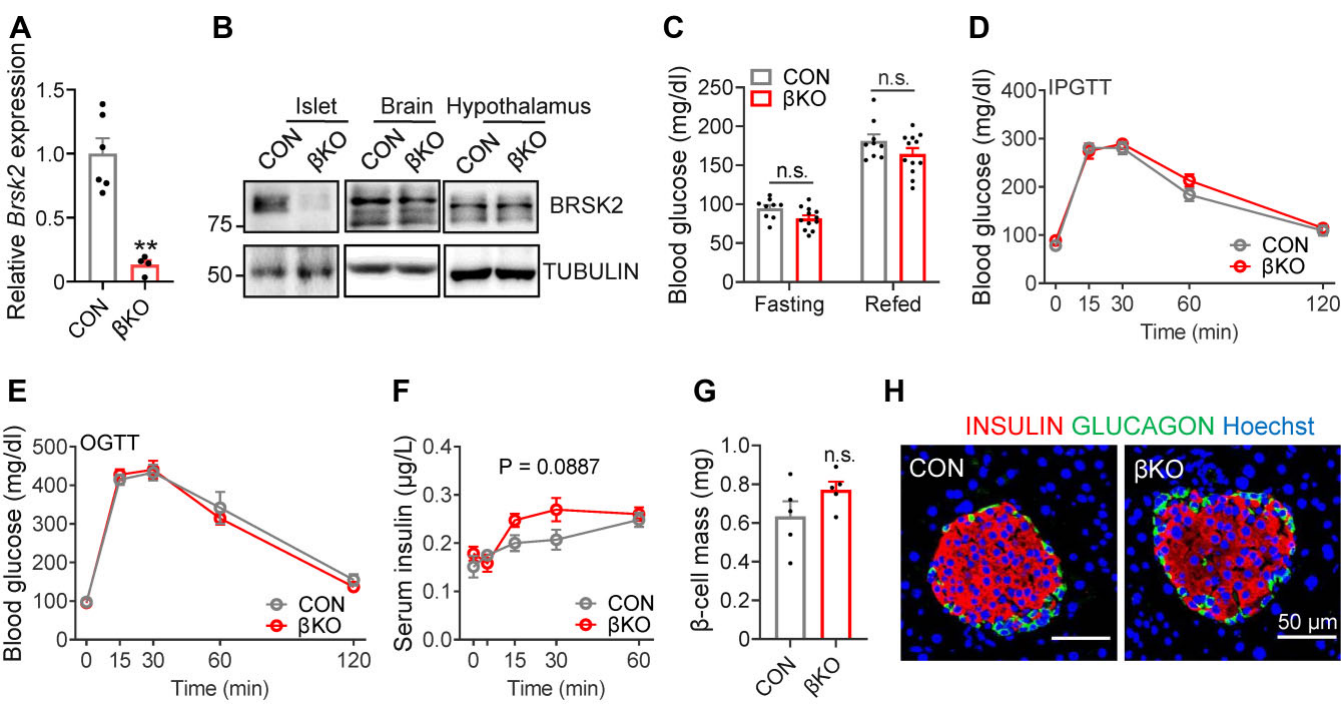
Loss-of-function BRSK2 in mature β cells has little effect on chow diet-fed mice. (**A**) qRT-PCR analysis of *Brsk2* in primary islets from CON and βKO mice 3 weeks after tamoxifen injection. *Actb* was used as an internal standard. (**B**) Western blot analysis of BRSK2 in primary islet, brain, and hypothalamus tissues from CON and βKO mice 3 weeks after tamoxifen injection. (**C**–**H**) Blood glucose levels at fasting and refed, during intraperitoneal glucuse tolerance tests (IPGTTs), and during OGTTs (**C**–**E**), serum insulin levels during IPGTTs (**F**), β-cell mass (**G**), and representative immunostaining images of INSULIN (red), GLUCAGON (green), and nuclei (Hoechst, blue) in pancreatic slices (**H**) of 3-month-old CON and βKO mice fed an chow diet. Data are presented as mean  ± SEM. *n* = 5–12 per group. n.s. = not significant.

Previous studies have shown that mice with global and pancreas-specific *Brsk2* deletion exhibited growth retardation, probably due to decreased serum insulin levels (Nie et al., 2013b, c). To avoid this developmental problem and to determine the physiological role of BRSK2 in β cells, we generated inducible β-cell-specific *Brsk2* knockout (βKO) mice (Supplementary Figure S3A). Since we previously demonstrated that both MIP-CreERT transgenic mice and Flox mice did not show any obvious abnormalities in glucose metabolism compared with wild-type (WT) mice (Liu et al., 2022), here, we used *Brsk2*^fl/fl^ mice injected with tamoxifen as the control (CON). Quantitative real-time polymerase chain reaction (qRT-PCR) results showed that *Brsk2* was specifically depleted in β cells but not in other tissues, such as the brain and hypothalamus (Figure 3A and B). When fed a normal chow diet, βKO mice were largely comparable to CON littermates, in terms of growth (Supplementary Figure S3B), fasting and refed blood glucose (Figure 3C), glucose excursion after *i.p.* and oral glucose injection (Figure 3D and E), β-cell and islet mass (Figure 3G; Supplementary Figure S3C), and the ratio of β cells to α cells (Figure 3H). However, serum insulin levels in βKO mice slightly increased (Figure 3F), indicating a high potential for insulin secretion.

**Figure 4:**
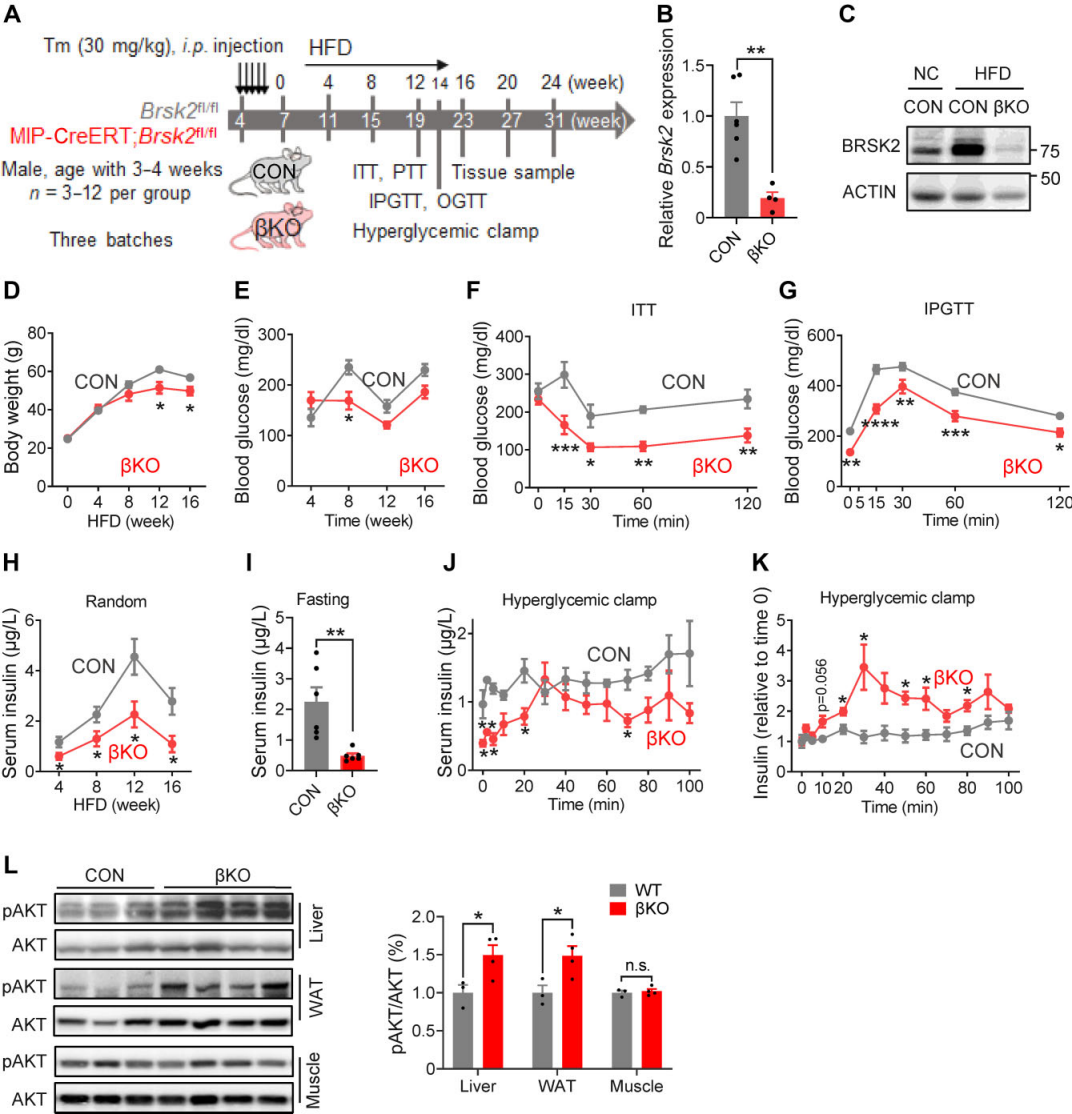
Loss-of-function BRSK2 in mature β cells improves systemic insulin sensitivity in mice. (**A**) Experimental scheme with CON and βKO mice. Tm, tamoxifen. (**B**) qRT-PCR analysis of *Brsk2* in mouse islets from βKO and CON mice fed an HFD for 12 weeks. *Actb* was used as an internal standard. (**C**) Western blot analysis of BRSK2 in mouse islets from βKO and CON mice fed a NC or HFD for 12 weeks. (**D**–**I**) Body weight (**D**) and random blood glucose (**E**) and insulin (**H**) levels in βKO and CON mice. (**F**) Insulin tolerance tests (ITTs) in 12-week HFD-fed mice showing blood glucose disposal by insulin. (**G**) IPGTTs in 14-week HFD-fed mice showing blood glucose levels at the indicated time. (**I**) Fasting serum insulin levels in βKO and CON mice fed an HFD for 12 weeks. (**J** and **K**) Insulin secretion (**J**) and relative insulin secretion adjusted with the basal insulin level per genotype (**K**) during hyperglycemic clamps in βKO and CON mice fed an HFD for 14 weeks. (**L**) Western blot analysis of pAKT(S308) levels at 30 min after *i.p.* injection of 1.5 g/kg glucose in the liver, WAT, and muscle of CON and βKO mice fed an HDF for 14 weeks, with quantitated pAKT/AKT shown on the right. Data are presented as mean ± SEM. *n* = 3–12 per group. n.s. = not significant, *P < 0.05, **P < 0.01.

When the mice were fed an HFD (Figure 4A), both protein and mRNA levels of BRSK2 in the islets remained significantly lower in βKO mice than in CON mice (Figure 4B and C). βKO mice initially gained body weight at a rate similar to that of CON littermates for the first 4 weeks, but the increasing rate slowed down significantly afterwards (Figure 4D). Random blood glucose levels were lower in βKO mice than in CON mice fed an HFD for 8 weeks (Figure 4E). Insulin sensitivity and glucose tolerance were greatly improved in βKO mice fed an HFD for 14 weeks (Figure 4F and G; Supplementary Figure S4A), with reduced serum insulin levels (Supplementary Figure S4B and C). Indeed, unlike the CON cohort, βKO mice fed an HFD did not develop hyperinsulinemia under either random or fasting conditions (Figure 4H and I). Furthermore, hyperglycemic clamp experiments showed that, while CON littermates fed an HFD were unresponsive to persistent hyperglycemia, βKO mice remained highly responsive to hyperglycemia-induced insulin secretion (Figure 4J and K; Supplementary Figure S4D). However, the islet mass and number of apoptotic β cells were not significantly changed in βKO mice (Supplementary Figure S4E and F).

The liver and white adipose tissue (WAT) of βKO mice fed an HFD remained highly insulin-sensitive. Hepatic glucose production from pyruvate was reduced in βKO mice fed an HFD (Supplementary Figure S4G). Phosphorylation of AKT at Ser308 was elevated in the liver and WAT but not in the skeletal muscle (Figure 4L). The expression levels of genes involved in *de novo* lipogenesis and inflammation were significantly reduced in the livers of βKO mice, while those of genes involved in gluconeogenesis were unchanged (Supplementary Figure S4H). Thus, our data demonstrated that BRSK2 deletion in mature β cells protected HFD-fed mice against hyperinsulinemia and insulin resistance.

### Gain-of-function BRSK2 in mature β cells leads to progressive diabetes in mice

**Figure 5:**
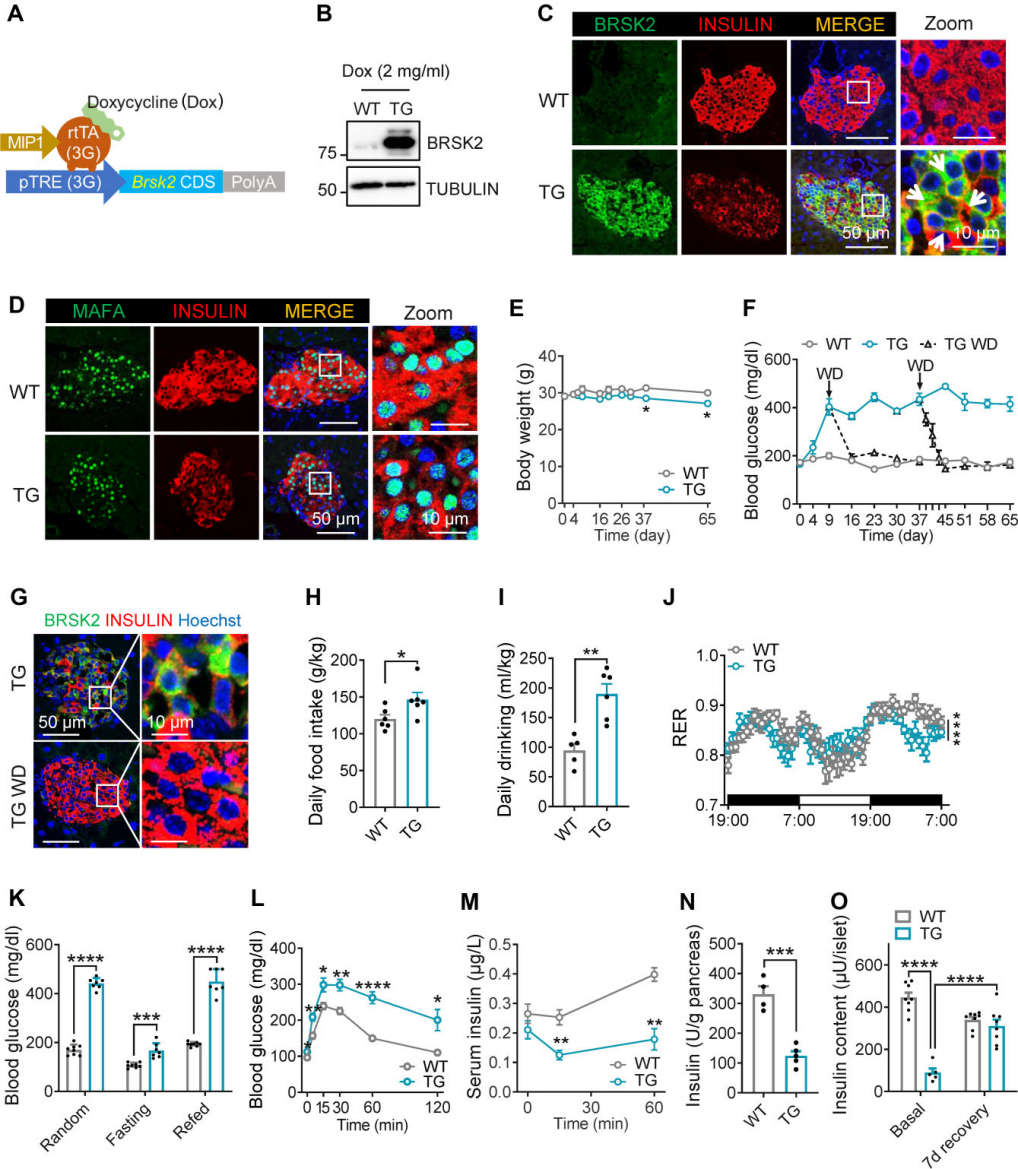
Gain-of-function BRSK2 in mature β cells leads to progressive diabetes in mice. (**A**) Schematic diagram of generating TG mice (MIP1-rtTA;*Brsk2*) using the Tet-On system. (**B**) Western blot analysis of BRSK2 in islets isolated from WT and TG mice with Dox induction for 7 days. (**C** and **D**) Representative immunofluorescence images of BRSK2 (**C**) and MAFA (**D**) in pancreatic slices from WT and TG mice with Dox induction for 7 days. (**E**) Body weights of WT and TG mice given drinking water with 2 mg/ml Dox for the indicated days. (**F**) Random blood glucose levels of WT and TG mice with Dox induction for the indicated time and subjected to Dox withdrawal (TG WD) on Day 9 or Day 37. (**G**) Representative images of BRSK2 and INSULIN in the pancreas of TG mice with Dox induction for 37 days and then with or without Dox for another 7 days. (**H**–**J**) Metabolic cage analysis showing daily food intake (**H**), daily drinking (**I**), and RER (**J**) of WT and TG mice with Dox induction for 4 weeks. (**K**) Random, 12-h fasting, and 2-h refed blood glucose levels in WT and TG mice with Dox induction for 4 weeks. (**L** and **M**) Blood glucose levels (**L**) and serum insulin levels (**M**) during IPGTTs in WT and TG mice with Dox induction for 4 weeks. (**N**) Insulin content in the whole pancreas of WT and TG mice with Dox induction for 4 weeks. (**O**) Insulin content in islets isolated from WT and TG mice with Dox induction for 4 weeks. Basal: 3 h after islet isolation; 7d recovery: isolated islets cultured in 5.5 mM glucose medium for 7 days. Data are presented as mean ± SEM. *n* = 4–8 per group. *P < 0.05, **P < 0.01, ***P < 0.001, ****P < 0.0001.

We next investigated the metabolic impact of *Brsk2* overexpression in β cells. Three independent lines of doxycycline (Dox)-inducible, β-cell-specific *Brsk2* transgenic (TG) mice (Figure 5A), Lines #20, #42, and #29, were established with similar metabolic phenotypes (not shown), excluding the possible artifacts caused by transgene insertion. The data from Line #20 are shown below. Dox administration only increased the BRSK2 protein level in islets (Figure 5B; Supplementary Figure S5A). Immunostaining revealed that BRSK2 was specifically expressed in INSULIN-positive β cells; however, there seemed a reverse correlation between BRSK2 and INSULIN expression levels (arrows, Figure 5C). To further verify the dose-dependent effect of BRSK2 on INSULIN expression, we repeated the immunostaining experiment following induction with different Dox doses. Indeed, β cells with higher BRSK2 expression exhibited lower expression of intracellular INSULIN (Supplementary Figure S5B). This effect was not due to β-cell dedifferentiation, transdifferentiation, or loss of β-cell number, as the immunostaining of β-cell maturation markers, such as MAFA and PDX1, and α/δ/PP-cell markers, the number of TUNEL-positive β cells, and β-cell mass were comparable between WT and TG littermates (Figure 5D; Supplementary Figure S5C–F).

After Dox induction, TG mice grew comparably to WT littermates initially and exhibited a modest decrease in body weight after 37 days (Figure 5E). However, TG mice developed progressive hyperglycemia over time, starting from Day 4, climbing to the peak (∼20 mM) on Day 9, and maintaining high glucose level during the Dox induction period (Figure 5F). This process was reversible, as after Dox withdrawal on Day 37, both the enhanced blood glucose level (Figure 5F) and the reduced INSULIN expression in β cells (Figure 5G), independent of alterations in insulin biosynthesis (Supplementary Figure S5G), returned to normal.

The observed body weight reduction in TG mice after Dox induction for 37 days was not due to the decrease in food intake, as TG mice increased food and drinking water intake after Dox induction for 4 weeks without interfering with feeding behavior, heat generation, or mouse activities (Figure 5H and I; Supplementary Figure S5H–K). TG mice favored using lipid as an energy source, as demonstrated by the reduced respiratory exchange ratio (RER) (Figure 5J), despite the significantly increased blood glucose levels under random–fasting–refed challenge (Figure 5K). IPGTT could not trigger an increase in insulin secretion after 4 weeks of Dox induction in TG mice, probably due to the dramatically reduced insulin levels in the pancreas (Figure 5L–N). Insulin content also decreased in the isolated islets from TG mice, which could be effectively recovered after *in vitro* culture in 5.5 mM glucose medium for 7 days (Figure 5O). These results suggest that BRSK2 overexpression in β cells progressively drives mouse diabetes progression from the initial stage to advanced stages.

### Gain-of-function BRSK2 in mature β cells leads to persistent hyperinsulinemia and systemic insulin resistance

**Figure 6:**
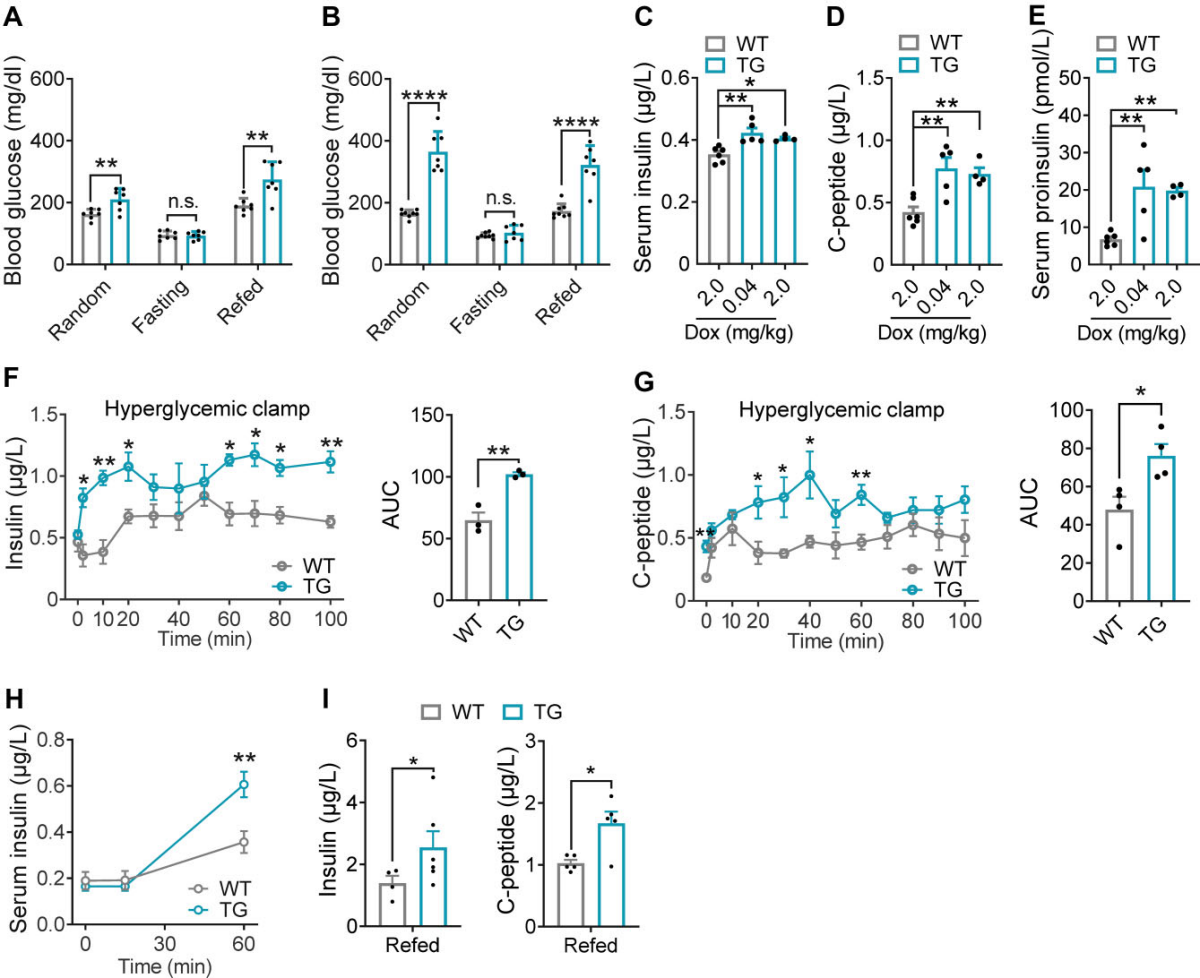
Gain-of-function BRSK2 in mature β cells leads to persistent hyperinsulinemia. (**A** and **B**) Random, 12-h fasting, and 2-h refed blood glucose levels in WT and TG mice with Dox induction for 4 days (**A**) or 2 weeks (**B**). (**C**–**E**) Serum insulin (**C**), C-peptide (**D**), and proinsulin (**E**) levels in WT and TG mice with the indicated Dox induction for 4 days. (**F** and **G**) Hyperglycemic clamps were performed in TG and WT mice with Dox induction for 4 days. Serum insulin (**F**) and C-peptide (**G**) levels at the indicated time, with calculated AUC shown on the right. Two independent batches of WT and TG mice were used for insulin and C-peptide determination. (**H**) Serum insulin levels during IPGTTs in WT and TG mice with Dox induction for 2 weeks. (**I**) Serum insulin and C-peptide levels after 2-h refed in WT and TG mice with Dox induction for 4 weeks. Data are presented as mean ± SEM. *n* = 4–8 per group. n.s. = not significant, *P < 0.05, **P < 0.01, ****P < 0.0001.

Then, we monitored blood glucose levels under random–fasting–refed challenge within 2 weeks of Dox induction. Although random and refed blood glucose levels in TG mice were significantly enhanced after 4 days or 2 weeks of Dox induction, fasting blood glucose levels were normal (Figure 6A and B). The hyperglycemia in TG mice did not result from absolute insufficiency of serum insulin levels, since circulating insulin, proinsulin, and C-peptide levels were significantly increased in TG mice after Dox administration (Figure 6C–E), indicating that BRSK2 might trigger β-cell hypersecretion.

Furthermore, glucose infusion provoked more insulin and C-peptide secretion in TG mice than in WT controls (Figure 6F and G; Supplementary Figure S6A and B). The hypersecretion of β cells was not associated with circulating GLP-1 or GIP levels (Supplementary Figure S6C and D). The hyperglycemic clamp results from TG mice confirmed the early alterations in HFD-fed mice. Indeed, one month of HFD feeding already increased the protein level of BRSK2 in islets, revealing a similar pattern of glucose-triggered insulin secretion (Supplementary Figure S6E–G). IPGTTs revealed more insulin secretion in TG mice after Dox induction for 2 weeks than in WT mice (Figure 6H). Surprisingly, while *i.p.* injection of glucose could not stimulate insulin secretion in TG mice after Dox induction for 4 weeks, refeeding was able to increase both insulin and C-peptide secretion (Figures 5M and 6I). Taken together, our data demonstrated that BRSK2 is both required and sufficient for hyperinsulinemia, likely in part via enhancing insulin secretion.

**Figure 7:**
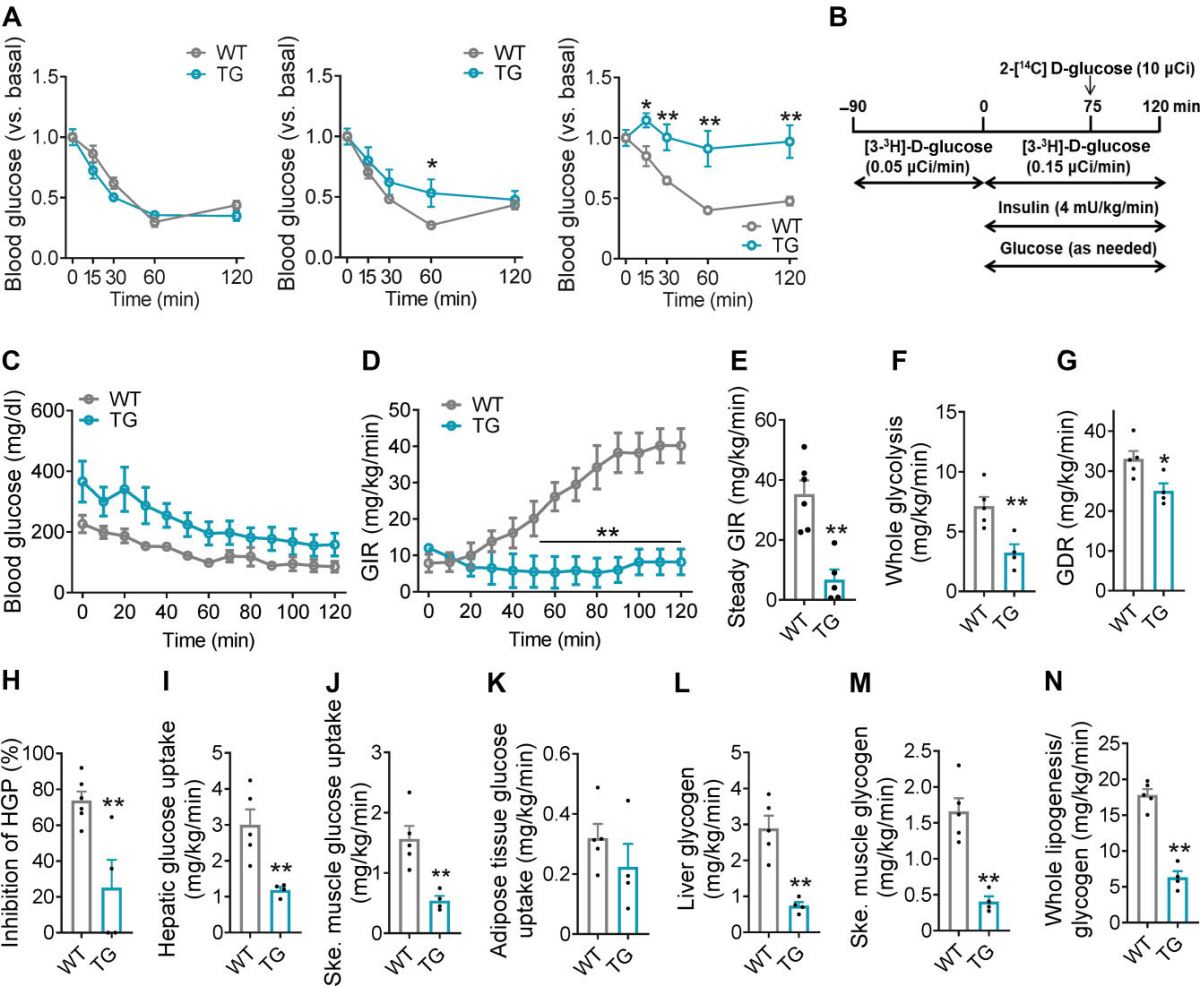
Gain-of-function BRSK2 in mature β cells leads to systemic insulin resistance. (**A**) Relative blood glucose levels by ITTs in WT and TG mice with Dox induction for 4 days (left), 2 weeks (middle), or 4 weeks (right). (**B**–**N**) Hyperinsulinemic–euglycemic clamps were performed in TG and WT mice with Dox induction for 1 week. (**B**) Schematic representation of the experimental procedure. Blood glucose level (**C**), GIR (**D**), steady GIR (**E**), whole glycolysis (**F**), GDR (**G**), HGP (**H**), hepatic glucose uptake (**I**), skeletal muscle glucose uptake (**J**), adipose tissue glucose uptake (**K**), synthesis of hepatic glycogen (**L**) and skeletal muscle glycogen (**M**), and whole lipogenesis/glycogenesis (**N**) during hyperinsulinemic–euglycemic clamps are shown. Data are presented as mean ± SEM. *n* = 5–8 per group. n.s. = not significant, *P < 0.05, **P < 0.01.

As persistent hyperinsulinemia often leads to insulin resistance (Templeman et al., 2017), we next tested insulin tolerance in these cohorts. While initially indistinguishable from WT mice, TG mice developed mild and severe insulin resistance after 2 and 4 weeks of DOX induction, respectively (Figure 7A). To explore the molecular mechanism underlying BRSK2-induced systemic insulin resistance, the hyperinsulinemic–euglycemic clamp assay was performed (Figure 7B). In response to a constant infusion of insulin, euglycemia was maintained by adjusting the infusion rate of glucose (Figure 7C). The glucose infusion rate (GIR) required to maintain euglycemia was much lower in TG mice (Figure 7D and E). Both glycolysis and the glucose disposal rate (GDR) were also reduced in TG mice (Figure 7F and G). Moreover, the inhibition of HGP by infused insulin was significantly attenuated in TG mice (Figure 7H). Glucose uptake was significantly decreased in the liver and skeletal muscle but not in the adipose tissue of TG mice (Figure 7I–K). Glycogen biosynthesis and lipogenesis were both reduced in TG mice (Figure 7L–N). Indeed, genes involved in *de novo* lipogenesis were significantly downregulated in both the WAT and liver of TG mice (Supplementary Figure S7A and B). The weights of subcutaneous WAT and epididymal WAT were much lower in TG mice than in WT mice due to the decreased size of adipocytes (Supplementary Figure S7C–E). Hepatic glycogen levels were also significantly reduced in TG mice (Supplementary Figure S7F). Hence, our data demonstrated that BRSK2 overexpression in β cells drives hyperinsulinemia and peripheral insulin resistance.

### Inhibition of BRSK2 promotes GSIS in a kinase-dependent manner in human islets

**Figure 8:**
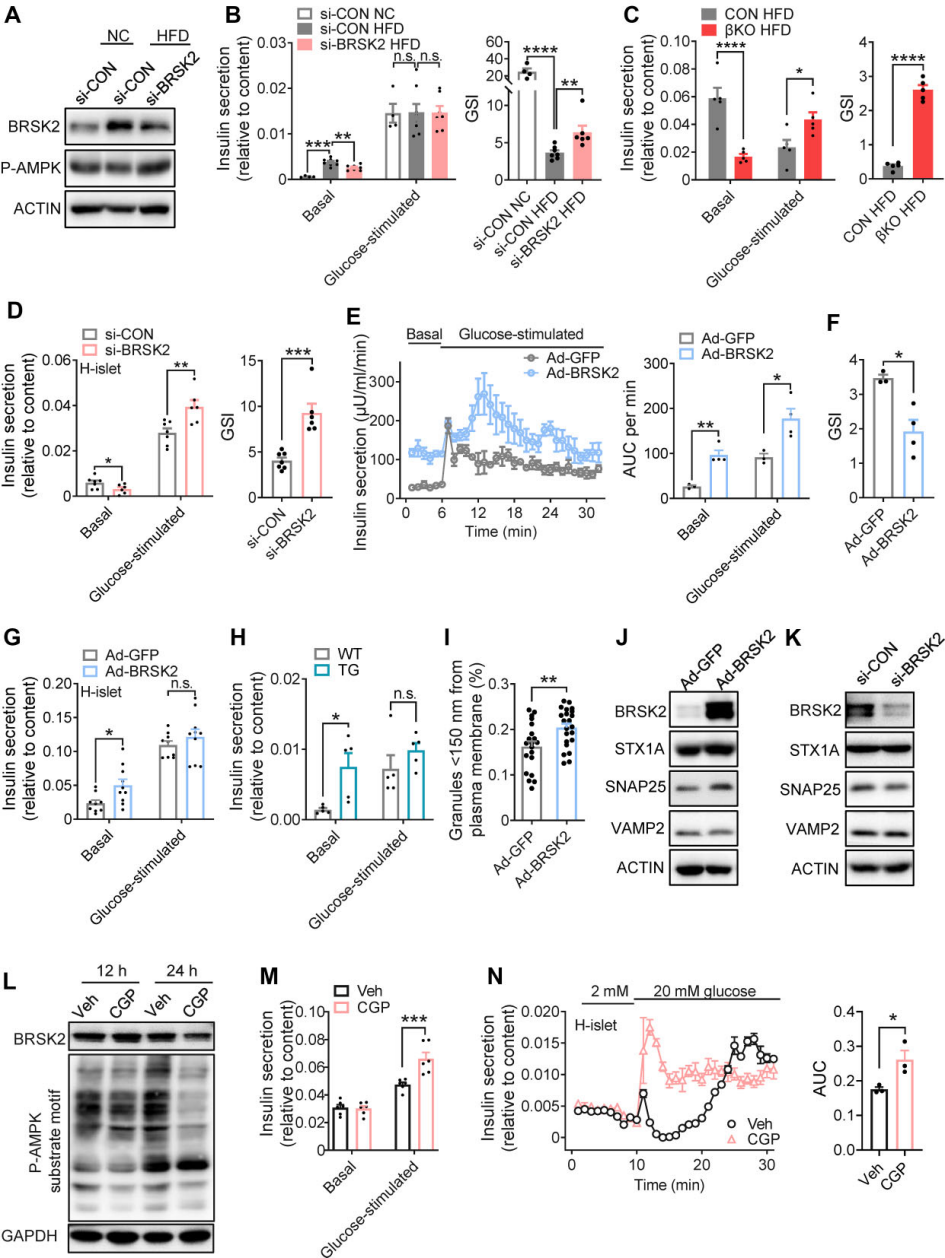
Inhibition of BRSK2 promotes GSIS in a kinase-dependent manner in human islets. (**A** and **B**) Primary islets isolated from mice fed a NC or HFD for 8 weeks were transfected with si-BRSK2 or si-CON for 48 h. (**A**) Western blot analysis of BRSK2 protein levels. (**B**) GSIS function shown as insulin secretion relative to content, with calculated GSI shown on the right. Basal = 3.3 mM glucose; glucose-stimulated = 16.7 mM glucose. (**C**) GSIS function of islets isolated from CON and βKO mice fed an HDF for 16 weeks, with calculated GSI shown on the right. (**D**) Human primary islets were transfected with si-BRSK2 or si-CON for 48 h and GSIS assays were performed, with calculated GSI shown on the right. (**E** and **F**) Mouse islets were infected with Ad-BRSK2 for 24 h. (**E**) Insulin secretion was determined by islet perfusion assays, with calculated AUC per minute shown on the right. (**F**) GSI was calculated as glucose-stimulated AUC divided by basal AUC. (**G**) Human islets were infected with Ad-BRSK2 for 24 h, and GSIS assays were performed. (**H**) GSIS assays in islets isolated from WT and TG mice with Dox induction for 7 days. (**I**) Ad-BRSK2-infected mouse islets were analyzed by TEM, and granules nearby the plasma membrane (<150 nm) were counted as membrane-fused insulin granules. Ad-GFP: *n* = 20; Ad-BRSK2: *n* = 22. (**J** and **K**) Western blot analysis of BRSK2, STX1A, SNAP25, and VAMP2 levels after Ad-BRSK2 infection for 24 h (**J**) or si-BRSK2 transfection for 48 h (**K**). ACTIN was used as an internal standard. (**L**) MIN6 cells were treated with CGP 57380 (10 μM) for the indicated time, and the protein levels of BRSK2 and P-AMPK substrate motif were detected. GAPDH was used as an internal standard. (**M**) MIN6 cells were treated with CGP 57380 (10 μM) for 24 h and GSIS assays were performed. (**N**) Islet perfusion assays were performed in human primary islets treated with or without CGP 57380 (10 μM) for 24 h (with calculated AUC of GSIS shown on the right). Data are presented as mean ± SEM. *n* = 3–10 per group. n.s. = not significant, *P < 0.05, **P < 0.01, ***P < 0.001, ****P < 0.0001.

Islet *Brsk2* knockdown significantly reduced basal insulin secretion during HFD feeding, thereby preserving glucose-sensing ability, as shown by the increased glucose-stimulated index (GSI) (Figure 8A and B). *Brsk2* knockdown also inhibited palmitate (Palm)-potentiated basal insulin secretion and rescued GSIS function in mouse islets (Supplementary Figure S8A and B). Furthermore, βKO mice were resistant to prolonged HFD-induced GSIS defects (Figure 8C). Moreover, knockdown of *BRSK2* in healthy human islets reduced basal insulin level but significantly enhanced glucose-stimulated insulin levels, thus showing a delightful GSI performance (Figure 8D).

Mouse islet perfusion assays showed that transfection with an adenovirus driving *BRSK2* overexpression increased both basal and glucose-stimulated insulin levels (Figure 8E), consistent with those in Nie et al. (2013b, c). However, GSI was decreased in mouse or human islets with *BRSK2* overexpression and primary islets from TG mice, due to enhanced basal insulin secretion (Figure 8F–H; Supplementary Figure S8C–G). Transmission electron microscopy (TEM) also showed increased membrane-fused insulin granules (Figure 8I; Supplementary Figure S8H), supporting the augmented basal insulin secretion by BRSK2. The effect of BRSK2 on insulin granule trafficking was assessed by using mOrange-NPY, which represents insulin. mOrange-NPY was localized near the plasma membrane in islet cells transfected with BRSK2 (Supplementary Figure S8I). The altered basal insulin secretion by BRSK2 was likely related to the t-SNARE but not the v-SNARE system. We found that two members of the t-SNARE family, STX1A and SNAP25, were positively regulated by BRSK2, while the v-SNARE family member VAMP2 was unchanged under both BRSK2 overexpression and knockdown conditions (Figure 8J and K).

To determine whether the effects of BRSK2 were kinase activity-dependent, we introduced a specific BRSK2 kinase inhibitor CGP 57380. As BRSK2 belongs to the AMPK family and shares a conserved kinase site (BRSK2 T174 phosphorylation site), treatment with CGP 57380 attenuated the phosphorylation of downstream AMPK substrates in β cells (Figure 8L), indicating the inhibition of BRSK2 kinase, but it did not affect cell viability (Supplementary Figure S8J). Inhibition of BRSK2 kinase activity by CGP 57380 enhanced insulin secretion in β cells in response to high-glucose stimulation (Figure 8M). Similarly, in human islet perfusion experiments, CGP 57380 treatment enhanced insulin secretion in response to 20 mM glucose stimulation (Figure 8N). The *ex vivo* results from human and mouse islets support that inhibition of BRSK2 could enhance GSIS capacity in a kinase-dependent manner.

## Discussion

Insulin is the only hormone that lowers blood glucose levels, and its synthesis and secretion by pancreatic β cells are harmonized by nutritional and gut hormonal signals in the context of intermittent feeding. Alterations in diet composition, such as a HFD, can promote insulin oversecretion and cause metabolic abnormities. In the present study, we demonstrated that BRSK2 links β-cell hypersecretion to diet-induced obesity, insulin resistance and T2DM. Large-scale human genetic data revealed the association of BRSK2 variants with human T2DM in the Chinese population. Moreover, in inducible genetic mouse models, we further showed that induction of BRSK2 expression in mature β cells caused persistent hyperinsulinemia, insulin resistance, and GSIS disability, while β-cell-specific *Brsk2* deletion prevented mice from HFD-induced obesity and glucose intolerance through preserved GSIS function and favorable insulin sensitivity. Our findings, for the first time, connect β-cell BRSK2 to T2DM pathogenesis in mice and probably in humans.

Our human study with a total of 6670 human genetic data revealed that three BRSK2 genetic variants rs112377266, rs61002819, and rs536028004 were significantly associated with worsening glucose metabolism and the onset of T2DM. These were distinct from the reported human BRSK2 variations related to neurodevelopmental disease (Hiatt et al., 2019). Specifically, rs112377266 might drive T2DM resulting from hyperinsulinemia, β-cell dysfunction, and insulin resistance (Figure 1A–D; Supplementary Figure S1A–D), while rs61002819 (Figure 1E and F; Supplementary Figure S1E–H) and rs536028004 (Figure 1G and H; Supplementary Figure S1I–L) merely connected insulin resistance with glucose intolerance. The hyperinsulinemia-coupled insulin resistance phenotypes in BRSK2 variant carriers recalled the hotly debated viewpoint that β cells may initiate the development and progression of T2DM via crosstalk with insulin-sensitive tissues, such as the liver, adipose tissue, and skeletal muscle (Sun et al., 2021). BRSK2 variant carriers may exhibit congenital hyperinsulinism due to β-cell hypersecretion, which causes a vicious cycle between hyperinsulinemia and insulin resistance, akin to the most recognized genetic variants of ABCC8 and KCNJ11 in T2DM (De Franco et al., 2020). As all three BRSK2 variants were not located in the coding region, the oversecretion of β cells is probably related to the alterations of BRSK2 protein levels in the cells. However, we could not distinguish whether the activating or inactivating genetic carriers of BRSK2 led to β-cell hypersecretion. Nevertheless, the increased BRSK2 protein level in human T2DM islets suggested that gain-of-function mutants of BRSK2 contribute to hyperinsulinemia-coupled insulin resistance.

Our study on genetic mouse models confirmed the effects of human BRSK2 variants. First, BRSK2 protein level was also enhanced in mouse islets within one month of HFD feeding, accompanied by hyperinsulinemia and overweight but euglycemia (Figure 2C; Supplementary Figure S2A–C). Second, deletion of *Brsk2* could protect mature mice from obesity, insulin resistance, and glucose intolerance by lowering insulin secretion and maintaining glucose-responsive insulin secretion under HFD conditions (Figure 4), raising the likelihood of an association between diabetes predisposition and BRSK2 levels in adults. Third, acute induction of BRSK2 expression in mature β cells caused severe hyperinsulinemia and mild hyperglycemia within a week (Figure 6), while chronic BRSK2 overexpression triggered frank diabetes characterized by insulin resistance (Figure 7) and GSIS disability (Figures 5M and 8H). Unexpectedly, regardless of whether BRSK2 was acutely or chronically induced, the mice recovered their normoglycemic state immediately after BRSK2 expression returned to baseline levels by using a Dox-inducible system (Figure 5F), suggesting that the amount of BRSK2 protein in β cells directly governs proper insulin secretion and glucose homeostasis.

Preclinical evidence has suggested that mild suppression of hyperinsulinemia can be a useful treatment strategy for obesity and insulin resistance (Page and Johnson, 2018), indicating hyperinsulinemia as a prerequisite for metabolic abnormities. Our hyperinsulinemic–euglycemic clamp results clearly showed that BRSK2-overexpressing mice exhibited decreased glucose uptake (Figure 7I–K) and hepatic glucose output (Figure 7H) and an inability to form adipose fat deposits (Supplementary Figure S7C–E), indicating systematic insulin resistance. The occurrence of HGP was attributed to an increased rate of hepatic glycogenesis/glycogenolysis and defects in *de novo* lipogenesis (Figure 7L–N). Indeed, gene expression levels in the liver and adipose tissue confirmed the inhibition of adipogenesis (Supplementary Figure S7A and B). These shrunk adipose tissues in BRSK2-overexpressing mice might be attributed to the acute insulin resistance caused by massive insulin secretion, consistent with the phenomenon caused by the insulin receptor antagonist S961 (Vikram and Jena, 2010; Aguayo-Mazzucato et al., 2019). The decreased hepatic glycogen levels in BRSK2-overexpressing mice (Figure 7L) were consistent with that observed in diabetic *db*/*db* mice and HFD-fed mice despite their high serum insulin levels (Sullivan et al., 2015; Mitchell et al., 2018).

Previous studies showed that mice with whole-body or pancreatic knockout of *Brks2* exhibit decreased β-cell size and islet mass (Nie et al., 2013c), suggesting that Brsk2 is important for islet development. In the present study, the islet mass in βKO mice was identical to that in CON littermates under both normal chow-diet (Figure 3G) and HFD (Supplementary Figure S4E) conditions, ruling out developmental defects and further extending our study to mature islets. Unlike the mice with global or pancreatic *Brsk2* deletion, which exhibit hypoinsulinemia and glucose intolerance (Nie et al., 2013c), mice with ablation of *Brsk2* in mature β cells exhibit slightly increased serum insulin levels in response to hyperglycemia under normal chow-diet condition (Figure 3F), indicating that the regulation of insulin secretion by BRSK2 involves more comprehensive secretory machineries of mature β cells (Nair et al., 2019; Sdao et al., 2021). Moreover, mature βKO mice were resistant to HFD-induced obesity and glucose intolerance by lowering basal insulin secretion and preserving GSIS capacity (Figures 4K and 8C). We also found that the increased basal insulin secretion in mice fed HDF or treated with Palm was reversed by Brsk2 knockdown (Supplementary Figure S8B). The *ex vivo* results prompted us to believe that BRSK2 was enrolled in sensing FFA, a well-recognized nutrient under the HFD condition (van Vliet et al., 2020; Corkey et al., 2021). Thus, we concluded that BRSK2 transduces lipid signals to induce basal insulin secretion, akin to the effects caused by the lipid receptor GRP40 (Steneberg et al., 2005; Wagner et al., 2013; Sabrautzki et al., 2017). Other cell autonomous regulators involved in controlling basal insulin secretion were also identified recently, e.g. ITGB1 (Yang et al., 2022). Our GSIS results from human primary islets also supported a cell autonomous role of BRSK2 in increasing basal insulin secretion (Figure 8D and G).

An increase in BRSK2 occurs prior to insulin resistance and hyperglycemia in HFD-fed mice, indicating an attempt to remodel β-cell function, islet mass, and architecture as part of a response to mild metabolic stress. However, islet adaptation would entail hyperinsulinemia, which promotes insulin resistance and β-cell exhaustion and inevitably leads to diabetes (Spanswick et al., 2000; Alarcon et al., 2016), as that occurs in BRSK2-overexpressing mice. Here, we found that BRSK2 in mature β cells did not contribute to such adaptations. However, immunostaining results from TG mice revealed a negative association between BRSK2 expression levels and the remaining insulin levels (Figure 5C), indicating that single β-cell hypersecretion contributes to hyperinsulinemia-induced insulin resistance. The reason for this selective alteration by BRSK2 was not clear, probably due to BRSK2-driven alterations in insulin secretion pathways, including insulin granule recruitment, docking, priming, and fusion (Omar-Hmeadi and Idevall-Hagren, 2021). It is well-known that v-SNAREs and t-SNAREs cooperate to modulate insulin secretion appropriately in response to fasting or feeding (Wu et al., 2017). Indeed, we found that t-SNAREs STX1A and SNAP25 were positively modulated by BRSK2, while v-SNARE VAMP2 was unchanged (Figure 8J and K), suggesting that the intact fusogenic SNARE machinery was enhanced in BRSK2-elevated β cells (Kang et al., 2022), similar to the findings in the early stage of *db*/*db* islets (Do et al., 2014). However, reductions in the SNARE machinery are associated with defective insulin secretion in overt T2DM patients and rodents, reminding us the defective GSIS function in BRSK2-overexpressing mice and improved GSIS function in *Brsk2* knockout mice. More detailed mechanisms of BRSK2-regulated GSIS capacity require further investigations in the *in vivo* mouse models with BRSK2 ablation or overexpression.

Alternatively, the kinase activity of BRSK2 at the conserved AMPK phosphorylation site also contributes to the enhanced insulin secretion. BRSK2 is mainly located at the membrane, where it forms AIS and KA1 tight structures at its C-terminus to bind to acidic phospholipids; BRSK2 therefore differs from AMPK in both location and function (Wu et al., 2015). Previous studies more focused on glucose- or GLP-1-regulated substrates, including GDIα and PAK1 (Nie et al., 2012, 2018). However, the specific substrate of BRSK2 involved in regulating basal insulin secretion has not been determined.

Like other AMPK family members, BRSK2 and its highly conserved BRSK1 isoform are two classic downstream targets of LKB1, and they establish neuronal polarity, axon growth, and neurotransmitter release under the control of LKB1 (Kishi et al., 2005; Barnes et al., 2007; Lilley et al., 2013). Mammalian LKB1 is also a critical regulator of cellular polarity in non-neural tissues, including β cells (Baas et al., 2004; Alessi et al., 2006; Rourke et al., 2018). Deletion of LKB1 in adult β cells alters β-cell polarity, which depends on the microtubule affinity-regulating kinase family, enlarges β-cell size, and enhances AMPK-dependent insulin secretion and content (Granot et al., 2009). The study did not include BRSK2 as a target because it focused on the biological functions of LKB1 in knockout mice rather than the pathological effects on disease development, e.g. diet-induced obesity and diabetes. Notably, the protein level of LKB1 was strongly increased in islets from both HFD-fed mice and genetic *leptin*-mutated *ob*/*ob* mice (Fu et al., 2009). We also observed a dramatic increase in BRSK2 in islets from HFD-fed mice (Figure 2C). Therefore, BRSK2 is more likely a metabolic response molecule that executes LKB1 function during diabetes onset.

In conclusion, human genetic data coupled with *in vivo* and *in vitro* experiments clearly indicate a critical role of BRSK2 in regulating GSIS function, subsequent systematic insulin resistance, and T2DM through crosstalk between β cells and insulin target tissues. We have provided definitive evidence that gain-of-function BRSK2 in β cells directly initiates β-cell hypersecretion, insulin resistance, and GSIS disability in mice, while loss-of-function BRSK2 in adulthood restrains diet-induced obesity and maintains GSIS function to prevent diabetes insult. These findings demonstrate that BRSK2 may be a viable therapeutic target for combating obesity and T2DM.

## Materials and methods

### Human study

Human subjects were drawn from Shanghai Nicheng Cohort Study. A detailed cohort study has been published before (Chen et al., 2018). All participants underwent OGTTs, and venous blood samples at 0, 30, and 120 min were collected to measure blood glucose and insulin levels. Insulin sensitivity and secretion were estimated by HOMA-IR, Gutt-ISI, and Stumvoll insulin secretion. The HOMA-IR is a measure of fasting insulin resistance and calculated using the following formula: [fasting glucose (mM)] × [fasting insulin (U/ml)]/22.5. The Gutt-ISI is a measure of post-glucose loading insulin resistance and calculated as: insulin sensitivity = *m*/(*G* × *I*), where *m* is a measure of glucose uptake during the OGTT and calculated from body weight and from fasting and 2-h glucose, *G* is the mean of fasting and 2-h glucose, and *I* is a log_10_ transformation of the mean of fasting and 2-h insulin. Thus, the unit for the Gutt-ISI is (mg × L)/(mmol × mU × min) (Kanauchi, 2002). Stumvoll 2^nd^ insulin secretion is defined as follows: 295 + 0.349 × insulin_60_ − 25.72 × glucose_60_ + 1.107 × insulin_0_. The single nucleotide polymorphisms (SNPs) in the *BRSK2* gene region were genotyped by Infinium Asian Screening Array and Infinium Multi-Ethnic Global BeadChip (Illumina). SNPs passing a quality-control procedure (individual call rate >98% and approved with Hardy–Weinberg equilibrium) were further imputed according to 504 East Asian subjects in the 1000 Genome Project Phase III, and SNPs with *R*-squared value >0.4 and minor allele frequency >0.01 were further analyzed. The statistical analysis was conducted with PLINK and SAS software (version 9.4; SAS Institute). Descriptive statistics were compared by *t*-test or non-parametric test according to data distribution. Genetic association analyses were performed by logistic and multiple linear regression adjusting for age and gender. ORs or β values were calculated according to the minor allele. This study was approved by the Human Research Ethics Committee of Shanghai Sixth People's Hospital Affiliated to Shanghai Jiao Tong University School of Medicine. Written informed consents were acquired from all subjects.

### Genetic mouse models

*Brsk2* ^fl/fl^ (The Jackson Laboratory, Cat#023199) and MIP-CreERT transgenic (The Jackson Laboratory, Cat#024709) C57BL/6J mice were purchased. MIP-CreERT;*Brsk2*^fl/fl^ mice, MIP-CreERT mice, and *Brsk2*^fl/fl^ mice (male, 3-week-old) were *i.p.* injected with tamoxifen (30 mg/kg, once a day) for 5 consecutive days to generate βKO mice and controls (CON). Mice were fed an HFD (Research Diets, D12492) or normal chow diet (D12450J) for the indicated months.

Dox-inducible, β-cell-specific *Brsk2* transgenic (TG) mice (MIP1-rtTA;*Brsk2*) were generated by the Model Animal Research Center of Nanjing University using the Tet-On system. MIP1-rtTA;*Brsk2* mice were inbred with C57BL/6J mice at least twice to obtain stable genetic offspring. Male mice aged 6–8 weeks were given drinking water containing Dox to induce BRSK2 overexpression *in vivo*.

All animal studies were performed according to guidelines established by the Research Animal Care Committee of Nanjing Medical University, China (permit number: IACUC-NJMU 1404075). Mice were housed at 23°C–25°C using a 12-h light/12-h dark cycle. Animals had *ad libitum* access to water at all times, and food was only withdrawn if required for an experiment. All experiments were performed in adult mice.

### Primary islet isolation and cell culture

Human islets were obtained from Tianjin First Central Hospital (Wang et al., 2019). The use of human islets was approved by the Research Ethics Committee of Nanjing Medical University. Murine islets were isolated as described previously (Zhu et al., 2013). Primary islets were cultured in medium (CMRL-1066 for human islets; RPMI-1640 for murine islets) containing 10% fetal bovine serume (FBS), 100 units/ml penicillin, and 100 mg/ml streptomycin. MIN6 cells (passages 21–35) were cultured in DMEM (Invitrogen) with 15% FBS (Gibco), 100 U/ml penicillin, 100 μg/ml streptomycin, 10 mM HEPES, and 50 μM β-mercaptoethanol (Sigma-Aldrich) (Miyazaki et al., 1990). The primary islets and MIN6 cells were incubated at 37°C in a suitable atmosphere containing 95% O_2_ and 5% CO_2_.

### Metabolic studies

Blood samples were collected from the tail vein and blood glucose levels were measured using a Glucometer Elite monitor (Abbott). Random blood glucose was measured at 9 AM in the morning. IPGTTs and OGTTs were performed by *i.p.* injection and oral gavage of D-glucose (1 g/kg), respectively, after overnight fasting. ITTs were performed by *i.p.* injection of 0.9 units/kg insulin after 4-h fasting. Pyruvate tolerance tests (PTTs) were performed by *i.p.* injection of 1.5 g/kg sodium pyruvate after 16-h fasting.

### Metabolic cage analysis

Mice were induced with Dox for 2 months and individually placed in a sterile metabolic cage unit (TSE Systems) for a multiday (5-day) study starting at 7 AM on Day 1. Standard 12-h light (7 AM–7 PM) and dark (7 PM–7 AM) cycles were maintained throughout the experiment. Mice were allowed to eat and drink *ad libitum*. Consumption of oxygen, exhalation of carbon dioxide, respiratory exchange rate, and activities were calculated and adjusted to body weight.

### Histology and immunostaining

Pancreas samples were rinsed in cold phosphate-buffered saline and fixed overnight in 4% paraformaldehyde. The samples were then processed and embedded in paraffin, and consecutive sections were incubated with primary antibodies (Supplementary Table S3), including anti-INSULIN (1:1000), anti-BRSK2 (1:100), anti-PDX1 (1:1000), anti-MAFA (1:100), anti-GLUCAGON (1:100), anti-somatostatin (1:100), and anti-pancreatic polypeptide (1:100) antibodies, followed by conjugated secondary antibodies (1:350). Images were captured and analyzed by a confocal laser scanning microscope (Olympus FV1200).

### Immunoblotting

Immunoblotting was performed with the corresponding antibodies (Supplementary Table S3) as previously described (Zhu et al., 2013).

### TEM

Purified pancreatic islets were fixed with 2.5% glutaraldehyde in 0.1 M sodium cacodylate buffer for 2 h and post-fixed in 1% OsO_4_, 1.5% K_4_Fe(CN)_6_, and 0.1 M sodium cacodylate for 1 h. Islets were *en bloc* stained, dehydrated, embedded, and cut into ultrathin sections (50–80 nm). The samples were visualized by a Tecnai Spirit Biotwin operated at 200 kV (FEI Company). At least 50 β cells were included for analysis.

### Plasmids, transfection, and infection

The human *BRSK2* overexpression plasmid pcDNA3.1-*BRSK2* and *BRSK2* adenoviruses were provided by Dr Yuguang Shi (University of Texas Health Science Center at San Antonio) (Nie et al., 2013b).

For siRNA-based interference assays, human and mouse primary islets and MIN6 cells were transfected with si-*BRSK2* or si-CON for 48 h. The si-*BRSK2* sequence was GGUUCGGGAACUUCAUCAA.

For adenovirus infection, 1.0 × 10^7^ pfu/ml human primary islets and 2.0 × 10^6^ pfu/ml MIN6 cells were infected with Ad-BRSK2 or Ad-GFP for 24 h.

### Islet perfusion and GSIS

For islet perfusion, 120 islets per group were incubated for 1 h at 37°C in Krebs–Ringer buffer (KRB) (135 mM NaCl, 3.62 mM KCl, 0.48 mM MgSO_4_·7H_2_O, 1.53 mM CaCl_2_, and 0.2% bovine serum albumin) with 2 mM glucose. Then, islets were collected in a syringe filter (Millex-GP; Millipore) for further perfusion: (i) perfusing with 37°C KRB with 2 mM glucose at 125 μl/min for 15 min to equilibrate; (ii) collecting the perfusate every minute for another 6 min; (iii) perfusing with 37°C KRB with 20 mM glucose for 25 min; and (iv) collecting the perfusate as previous. Then, 7–12 min was considered the first phase of insulin secretion, while 12–22 min was considered the second phase of insulin release.

For GSIS assays, islets or MIN6 cells were incubated in KRB for 1 h, followed by low-glucose (2 mM for MIN6 cells; 3.3 mM for primary islets) incubation for 1 h and then high-glucose (20 mM for MIN6 cells; 16.7 mM for primary islets) incubation for another 1 h. The supernatants were collected for measurement of insulin secretion. Cellular, islet, and pancreatic insulin contents were extracted using an acid–ethanol solution (0.15 M HCl in 75% ethanol in H_2_O) overnight at 4°C. Insulin levels in supernatants and insulin contents were measured by radioimmunoassay as previously described and normalized to total cellular DNA levels or the weight of the pancreas.

### Hyperinsulinemic–euglycemic clamps

Mice were anesthetized by isoflurane inhalation, and right internal jugular vein catheterization was performed in a sterile environment. Mice were allowed a 5-day postsurgical recovery and those with <5% weight loss were subsequently studied. After measurement of fasting blood glucose level and body weight, the mice were carefully placed in a mouse fixator for a few minutes to equilibrate. Mice were equilibrated from *t* = −90 to 0 min after 4–6 h of fasting. [3-^3^H] glucose (3 μCi; Moravek) was administered at *t* = −90 min, followed by a constant infusion of 0.05 μCi/min. After the basal period (*t* = −90–0 min), blood samples were collected from the tail vein to determine the plasma glucose concentration and basal glucose-specific activity. Then, continuous human insulin (Humulin; Novo Nordisk) infusion started (*t* = 0 min) at a rate of 4 mU/kg/min to maintain the hyperinsulinemic condition with submaximal suppression of HGP to assess insulin sensitivity. At 0 min, the continuous infusion rate of the [3-^3^H]-D-glucose tracer was increased to 0.15 μCi/min to minimize the change in glucose-specific activity. At *t* = 75 min, 2-[^14^C] D-glucose (10 μCi; Moravek) was administered into each mouse to measure glucose uptake. Blood samples were collected at 10-min intervals from the tail vein, and blood glucose concentrations were measured with a glucose meter. During 120-min clamping, variant glucose was simultaneously infused to keep the blood glucose concentration stable (∼130 mg/dl). GIR was recorded to assess insulin sensitivity. At the end, additional blood samples were collected to determine the plasma glucose concentration and glucose-specific activity. The liver, muscle, and WAT were collected for the determination of radioactivity. Serum and tissue radioactivity were measured and calculated according to Kim (2009).

### Hyperglycemic clamps

Mice were anesthetized by isoflurane inhalation after overnight fasting and subjected to a 2-h hyperglycemic clamp assay with a continuous infusion of 50% glucose at an adjusted rate to sustain the blood glucose level at ∼400 mg/dl. GIR and blood glucose and serum insulin levels were recorded at 0, 2, 5, 10, and every 10 min until 120 min. Then, 0–20 min was considered the first phase of insulin secretion, while 20–120 min was considered the second phase of insulin release.

### qRT-PCR

Total RNA was extracted using TRIzol reagent (Invitrogen), and cDNA synthesis was carried out using the HiScript II Q RT SuperMix (Vazyme). qRT-PCR was performed by using SYBR qPCR Master Mix (Vazyme) on the LightCycle 480 Instrument II (Roche). Gene expression levels were normalized to that of *Actb* or *Arpppo*. Primer sequences for qRT-PCR are listed in Supplementary Table S4.

### Statistical analysis

Data from three independent experiments and carried out in triplicate were combined, and the results were presented as mean ± SEM. Statistical differences were assessed using Prism 9 (GraphPad Software). Unpaired two-tailed Student's *t*-test and two-way ANOVA were used. *P*< 0.05 was considered statistically significant.

## Supplementary Material

mjad033_Supplemental_File
