# Supplementary material for: BRSK2 in pancreatic β cells promotes hyperinsulinemia-coupled insulin resistance and its genetic variants are associated with human type 2 diabetes
Source: J Mol Cell Biol. 2023 May 15;15(5):mjad033. doi: 10.1093/jmcb/mjad033 (PMC10782904; doi:10.1093/jmcb/mjad033)
Supplement: mjad033_Supplemental_File [file mjad033_supplemental_file.pdf]

Supplementary material

BRSK2 in pancreatic β cells promotes hyperinsulinemia-coupled insulin resistance and its genetic variants are associated with human type 2 diabetes

Running title: β-cell BRSK2 leads to T2DM

Rufeng Xu[1], Kaiyuan Wang[1], Zhengjian Yao[1], Yan Zhang[1], Li Jin[2,6], Jing Pang[1], Yuncai Zhou[1], Kai Wang[1], Dechen Liu[1], Yaqin Zhang[1], Peng Sun[1], Fuqiang Wang[3], Xiaoai Chang[1], Tengli Liu[4], Shusen Wang[4], Yalin Zhang[5], Shuyong Lin[5], Cheng Hu[2,6,*], Yunxia Zhu[1,*], and Xiao Han[1,*]

[1] Key Laboratory of Human Functional Genomics of Jiangsu Province, Nanjing Medical University, Nanjing 211166, China

[2] Institute for Metabolic Disease, Fengxian Central Hospital Affiliated to Southern Medical University, Shanghai 201499, China

[3] Analysis Center, Nanjing Medical University, Nanjing 210029, China

[4] Organ Transplant Center, Tianjin First Central Hospital, Nankai University, Tianjin 300192, China

[5] State Key Laboratory for Cellular Stress Biology, School of Life Sciences, Xiamen University, Xiamen 361102, China

[6] Shanghai Diabetes Institute, Shanghai Key Laboratory of Diabetes Mellitus, Shanghai Clinical Center for Diabetes, Shanghai Sixth People's Hospital Affiliated to Shanghai Jiao Tong University School of Medicine, Shanghai 200233, China

* Correspondence to: Xiao Han, E-mail: hanxiao@njmu.edu.cn; Yunxia Zhu, E-mail: zhuyx@njmu.edu.cn; Cheng Hu, E-mail: alfredhc@sjtu.edu.cn

Supplementary Figures

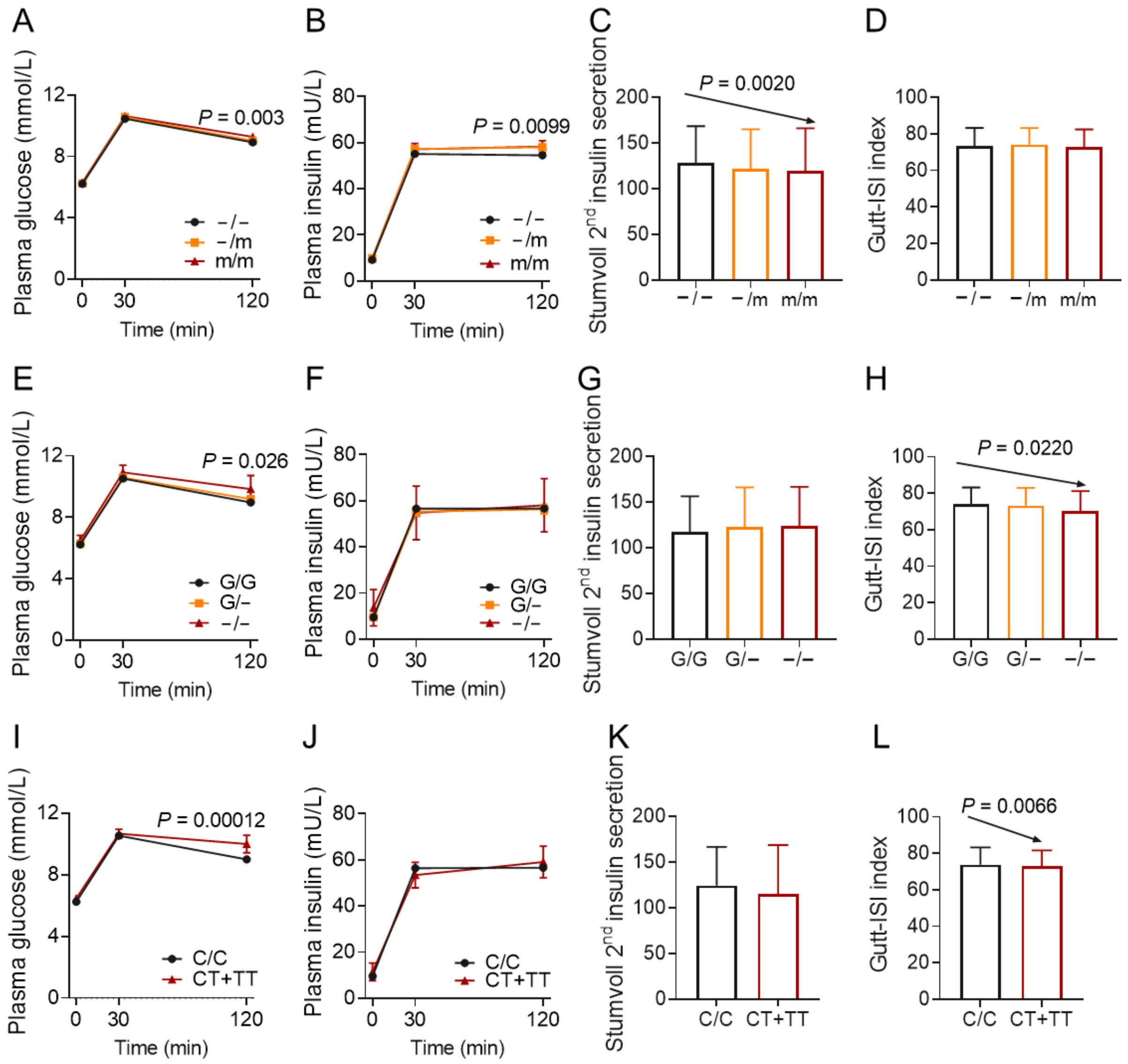

Supplementary Figure S1. Association between BRSK2 and glucose metabolism in humans, related to Figure 1. (**A**–**L**) Brsk2 loci genotyping of rs112377266 (**A**–**D**), rs61002819 (**E**–**H**), and rs536028004 (**I**–**L**). Each panel (from left to right) showed the plasma glucose level (**A**, **E**, **I**), insulin level (**B**, **F**, **J**), Stumvoll 2nd insulin secretion (**C**, **G**, **K**), and Gutt insulin sensitivity index (Gutt-ISI, **D**, **H**, **L**) after OGTT.

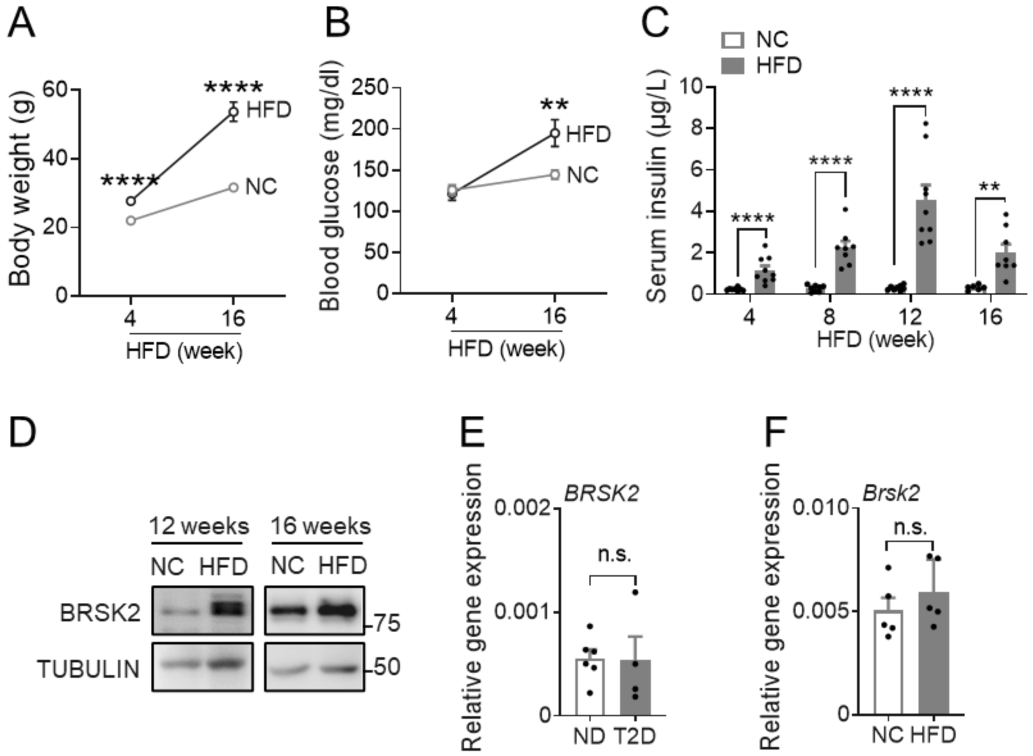

Supplementary Figure S2. Elevated BRSK2 level in islet β cells is associated with obesity and hyperinsulinemia, related to Figure 2. (**A**–**C**) Body weight (**A**), fasting blood glucose levels (**B**), and serum insulin levels (**C**) in wildtype C57BL/6J mice fed on NC or HFD for 4–16 weeks. (**D**) Western blot analysis of BRSK2 in primary mouse islets from mice fed on NC and HFD for 12 weeks or 16 weeks (n = 3 mice per group). (**E**) qRT-PCR analysis of *BRSK2* gene expression in primary human islets from non-diabetic and T2-diabetic subjects (n = 6 for ND, n = 4 for T2D). (**F**) qRT-PCR analysis of *Brsk2* gene expression in primary islets from NC and HFD mice (n = 5 per group). Data were presented by Mean ± SEM. n.s. = not significant, ***P* < 0.01, *****P* < 0.0001.

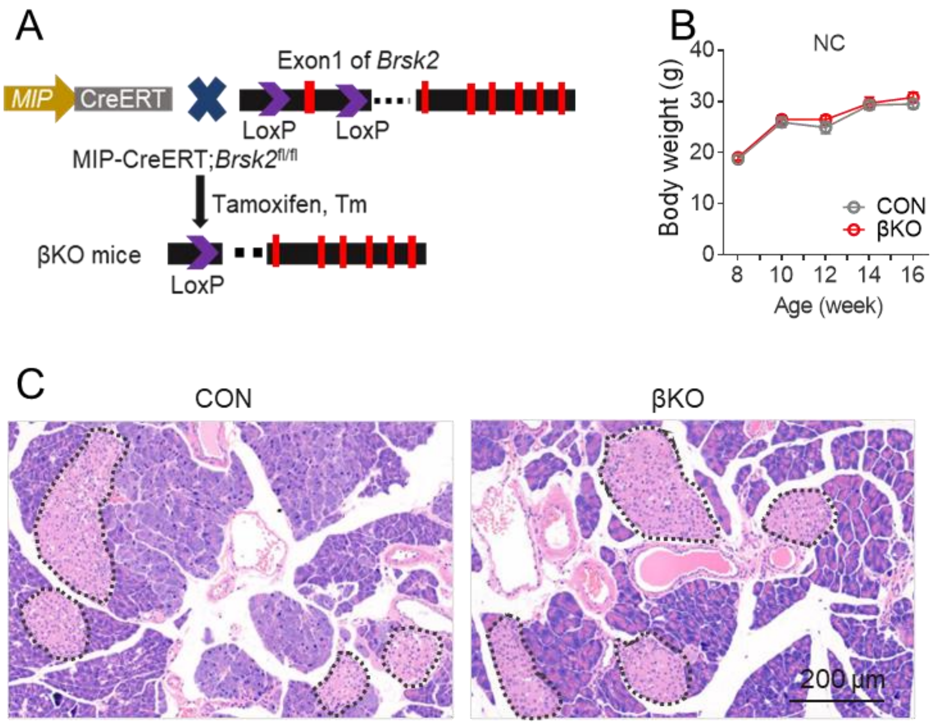

Supplementary Figure S3. Deletion of BRSK2 in mature β cells has no effect on mouse growth, related to Figure 3. (**A**) Schematic of β-cell-specific *Brsk2* knockout (βKO) mice. Cre-negative mice with tamoxifen (Tm) injection were served as control (CON) group. (**B**) Body weights of βKO and CON mice under normal chow-diet (NC) condition. (**C**) Representative H&E images of pancreatic slices showing islet area of CON and βKO mice at 3-month age fed on NC. Data were presented by Mean ± SEM. n = 4–8 per group.

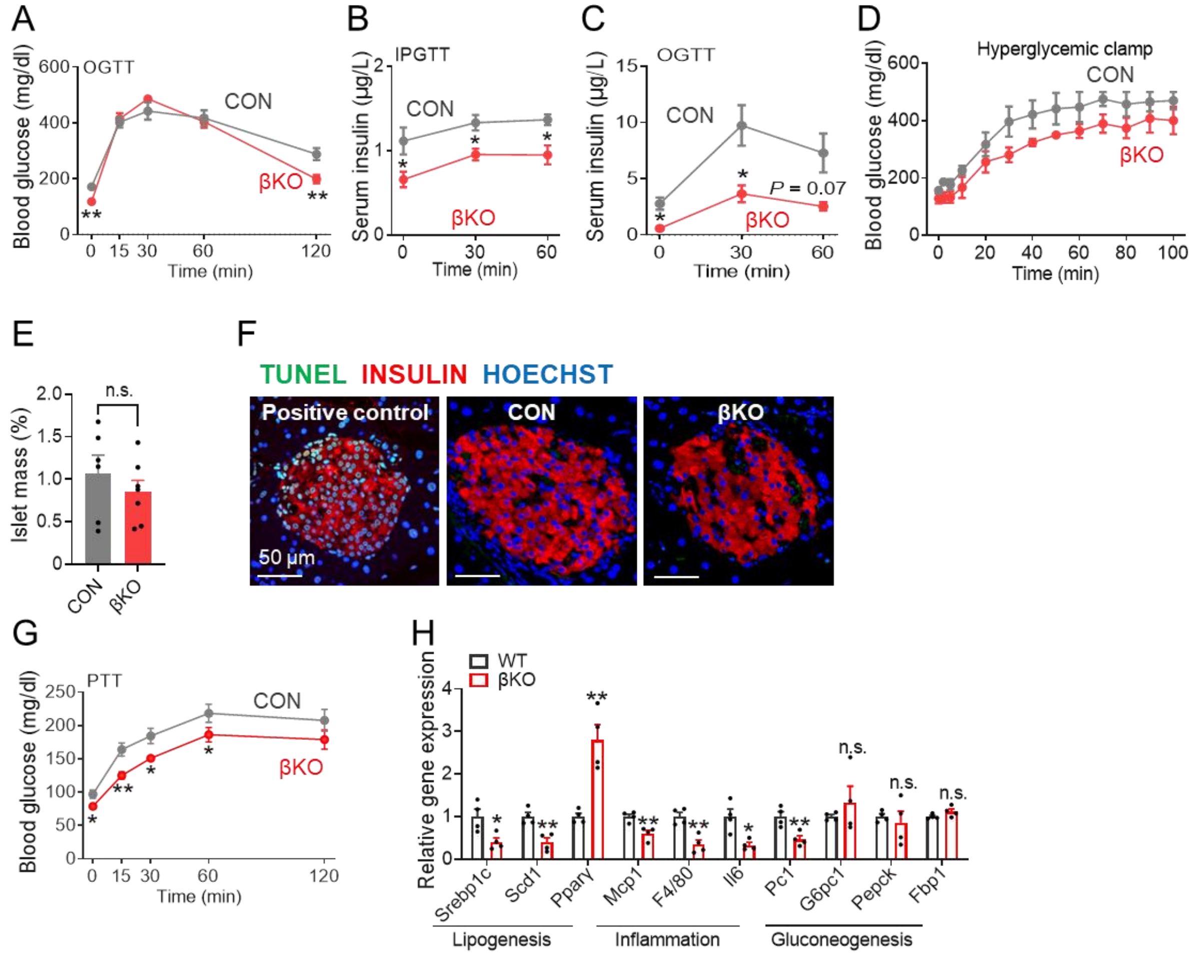

Supplementary Figure S4. βKO improves HFD-induced metabolic abnormities in mice, related to Figure 4. (**A**–**C**) Blood glucose levels during OGTTs (**A**) and serum insulin levels during IPGTTs (**B**) and OGTTs (**C**) in CON and βKO mice with HFD feeding for 14 weeks. (**D**) Blood glucose levels during hyperglycemic clamps in CON and βKO mice with HFD feeding for 14 weeks. (**E**) Islet mass in CON and βKO mice with HFD feeding for 14 weeks. (**F**) Representative images of TUNEL (green) and INSULIN (red) staining in CON and βKO mice with HFD feeding for 14 weeks. Nuclei (blue). (**G**) Levels of blood glucose during pyruvate tolerance tests (PTTs) in CON and βKO mice with HFD feeding for 12 weeks. (**H**) Relative gene expression levels in the livers from CON and βKO mice with HFD feeding for 14 weeks. Data were presented by Mean ± SEM. n = 4–8 per group. n.s. = not significant, **P* < 0.05, ***P* < 0.01.

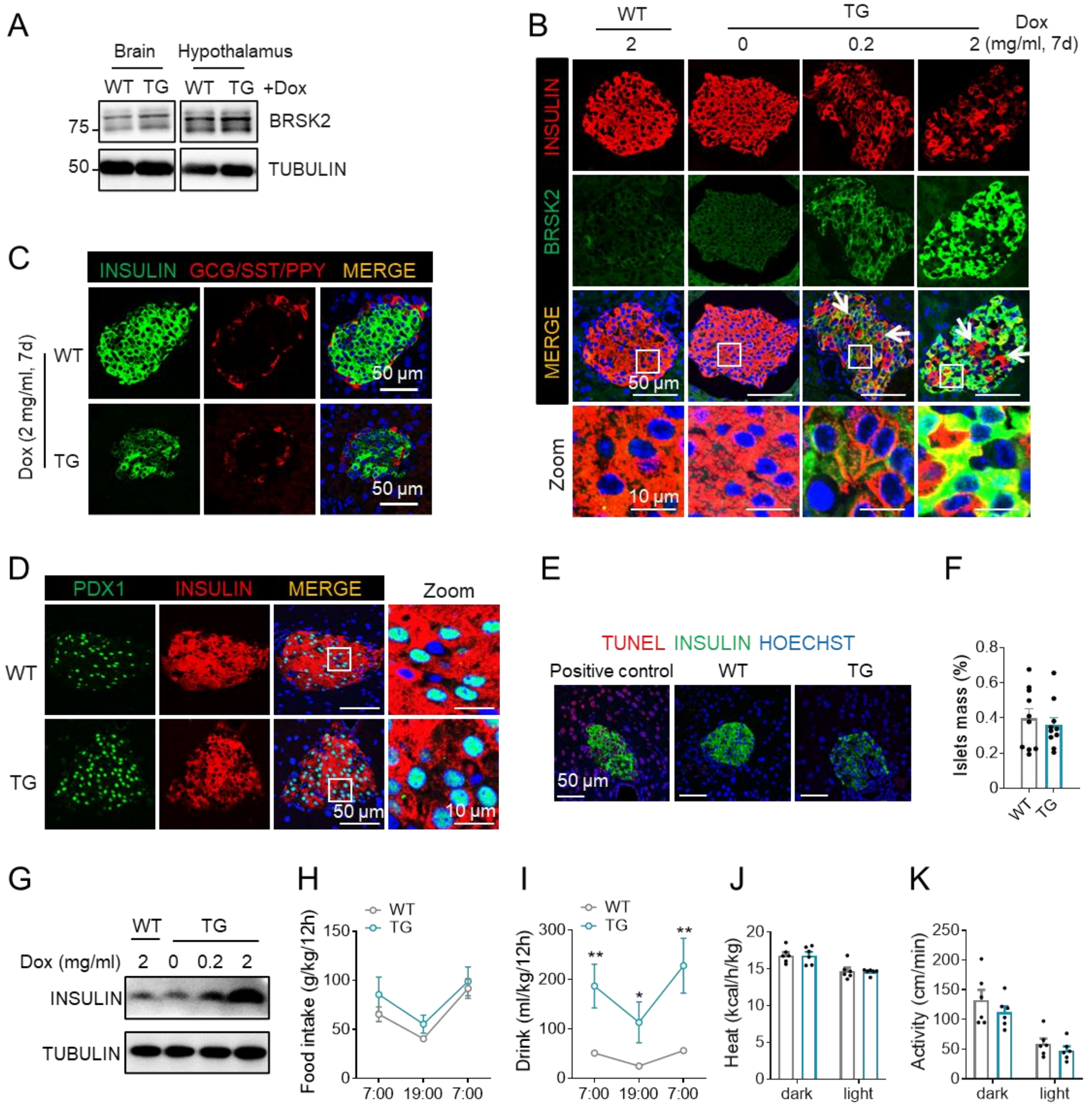

Supplementary Figure S5. Gain-of-function of BRSK2 in β cells reduces cellular INSULIN amount in a BRSK2-dose dependent manner, related to Figure 5. (**A**) Western blot analysis of BRSK2 protein levels in brain and hypothalamus from WT and TG mice with Dox induction for 7 days. TUBULIN was used as an internal standard. (**B**) Representative images of BRSK2 (green) and INSULIN (red) in pancreas slices from WT and TG mice with indicated concentrations of Dox induction for 4 days. (**C**) Representative images of INSULIN (green) and glucagon+somatostatin+pancreatic polypeptide (GCG/SST/PPY, red) in pancreas obtained from WT and TG mice with Dox induction for 7 days. (**D**) Representative images of PDX1 (green) and INSULIN (red) in pancreas obtained from WT and TG mice with Dox induction for 7 days. (**E**) Representative images of TUNEL (green) and INSULIN (red) in pancreas obtained from WT and TG mice with Dox induction for 30 days. (**F**) Islet mass in WT and TG mice with Dox induction for 30 days. (**G**) Western blot analysis of INSULIN protein levels in islets from WT and TG mice with Dox induction for 4 days. TUBULIN was used as an internal standard. (**H**–**K**) Metabolic cage showing day–night food intake (**H**), drinking water (**I**), heat (**J**), and activities (**K**) of WT and TG mice with Dox induction for 4 weeks. Data were presented by Mean ± SEM. n = 5–10 per group. $^{**}P < 0.01$.

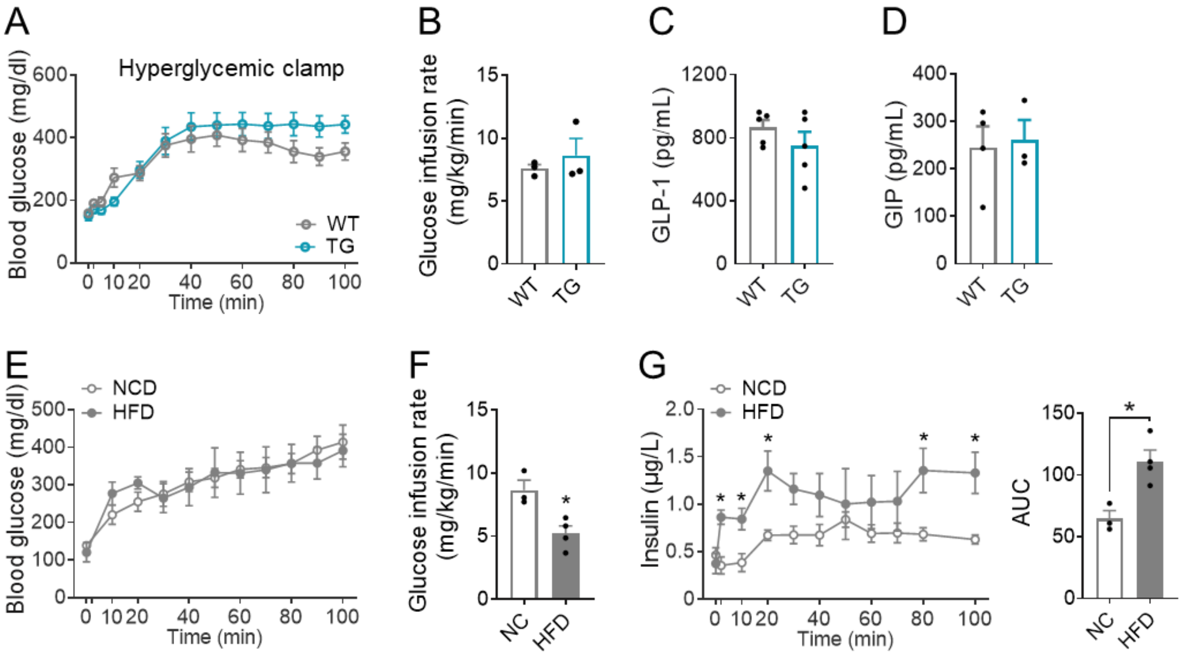

Supplementary Figure S6. Hyperglycemic clamps in TG mice and HFD-fed mice, related to Figure 6. (**A** and **B**) Blood glucose levels (**A**) and glucose infusion rate (**B**) during hyperglycemic clamps in WT and TG mice with Dox induction for 4 days. (**C** and **D**) Serum levels of GLP-1 (**C**) and GIP (**D**) of WT and TG mice with Dox induction for 7 days. (**E**–**G**) Blood glucose levels (**E**), glucose infusion rate (**F**), and serum insulin levels (**G**, quantitated AUC on the right) during hyperglycemic clamps in NC and 4-week–HFD-fed mice. Data were presented by Mean ± SEM. n = 3–5 per group. *$P < 0.05$.

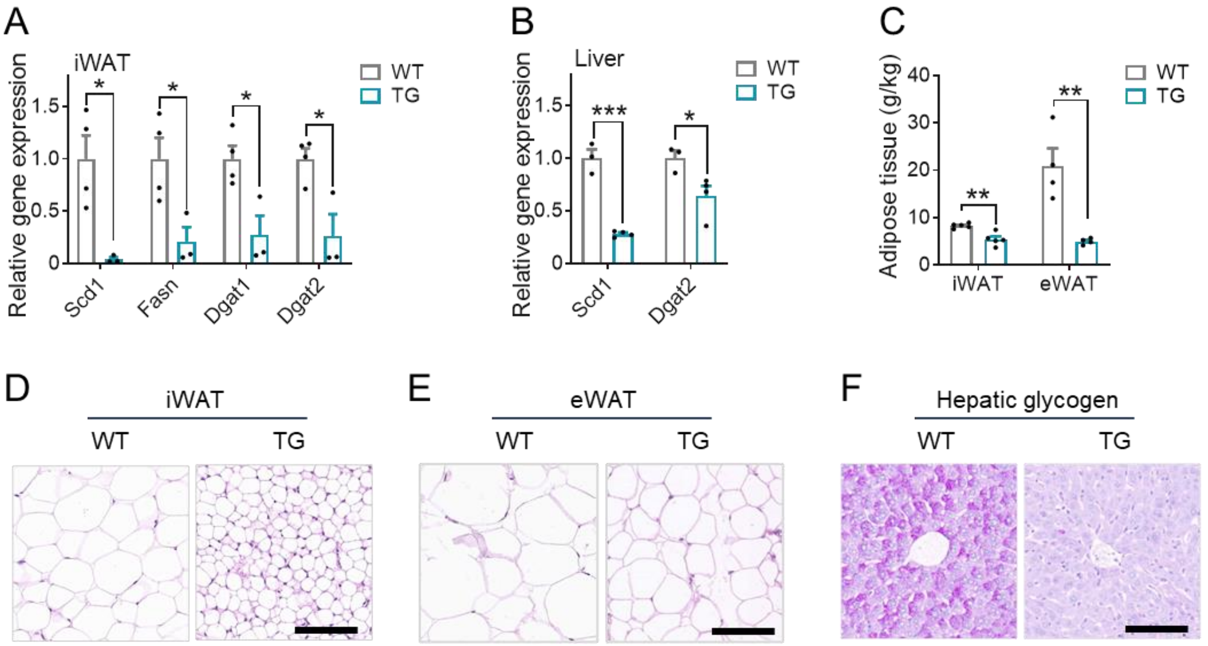

Supplementary Figure S7. Gene expressions and morphological alterations in TG mice, related to Figure 7. (**A** and **B**) qRT-PCR analysis of lipogenesis genes in iWAT (**A**) and liver tissues (**B**) from WT and TG mice with Dox induction for 2 weeks. *Actb* was used as an internal control. (**C**) Weights of adipose tissues relative to body weights in WT and TG mice with Dox induction for 2 weeks. (**D** and **E**) Representative H&E images of iWAT (**D**) and eWAT (**E**) showing adipocyte size in WT and TG mice with Dox induction for 2 weeks. Bar = 100 μm. (**F**) Periodic acid–Schiff (PAS) staining showed the glycogen levels in the livers of WT and TG mice with Dox induction for 2 weeks. Bar = 100 μm. Data were presented by Mean ± SEM. n = 4 per group. n.s. = not significant, **$P < 0.01$, ***$P < 0.001$, ****$P < 0.0001$.

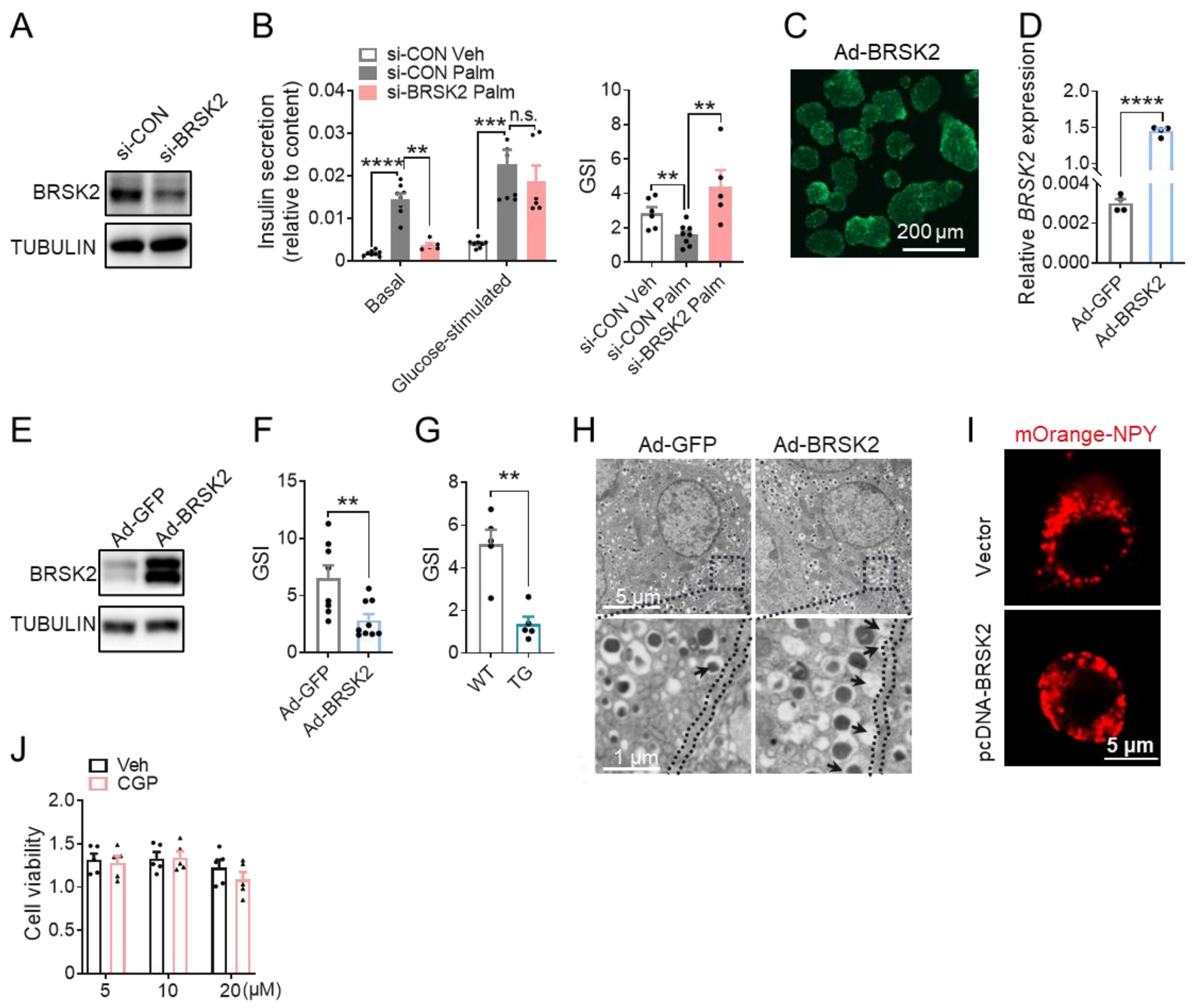

Supplementary Figure S8. BRSK2 enhances basal insulin secretion, related to Figure 8. (**A** and **B**) Primary mouse islets were transfected with si-BRSK2 and si-CON for 48 h. (**A**) Western blot analysis of BRSK2 levels to show interference efficiency. (**B**) GSIS function was showed as relative to insulin content, with quantitated GSI on the right. Basal = 3.3 mmol/L; Glucose-stimulated = 16.7 mmol/L glucose. (**C**) Representative images of human islets infected with Ad-BRSK2 for 24 h. (**D**) qRT-PCR analysis of *BRSK2* level in human islets infected with Ad-GFP or Ad-BRSK2 for 24 h. *ACTB* was used as an internal control. (**E**) Western blot analysis of BRSK2 protein levels in mouse islets infected with Ad-GFP or Ad-BRSK2 for 24 h. TUBULIN was used as an internal standard. (**F**) GSI quantitation of human islets infected with Ad-BRSK2 for 24 h. (**G**) GSI quantitation of islets isolated from WT and TG mice with Dox induction for 7 days. (**H**) Transmission electron microscopy (TEM) showed membrane-fused insulin granules in mouse islets infected with Ad-GFP or Ad-BRSK2 for 24 h. (**I**) Representative images of mOrange-NPY in MIN6 cells co-transfected with pcDNA-BRSK2 for 24 h. (**J**) MIN6 cells were treated with indicated doses of CGP 57380 for 48 h and CCK8 assays were performed to detect cell viabilities. Data were presented by Mean ± SEM. n = 4 per group. n.s. = not significant, *$P < 0.05$, **$P < 0.01$, ***$P < 0.001$.

Supplementary Tables

Supplementary Table S1. Clinical characteristics.

	**Control**	**T2DM**
N	4223	2447
Age (year)	55.71±7.11	58.54±6.91
Gender (male%)	0.41	0.41
BMI (kg/m^2)	24.25±3.02	26.03±3.32
Body fat (kg)	29.2(22.5,35)	32.2(25.4,38.3)
WC (cm)	82(76,88)	88(82,94)
WHR	0.88(0.84,0.92)	0.92(0.88,0.96)
SBP (mmHg)	130(120,140)	138(128,150)
DBP (mmHg)	82(78,90)	84(80,90)
Uric acid (μmol/L)	284(238,336)	298(251,355)
Total cholesterol (mmol/L)	4.95(4.39,5.59)	5.24(4.62,5.91)
Triglyceride (mmol/L)	1.17(0.84,1.69)	1.65(1.15,2.45)
Low-density lipoprotein (mmol/L)	2.98(2.5,3.48)	3.17(2.66,3.73)
High-density lipoprotein (mmol/L)	1.29(1.08,1.53)	1.21(1.03,1.42)
Free fatty acid (μmol/L)	453(342,582)	659.5(504,838)
HbA1c (%)	5.5(5.2,5.7)	6.5(5.9,7.4)
Fasting plasma glucose (mmol/L)	5.51(5.23,5.76)	7.41(6.63,8.79)
PG30 (mmol/L)	9.21(8.22,10.2)	12.26(10.74,14.12)
PG2H (mmol/L)	6.36(5.53,7.05)	14.16(11.91,17.61)
Fasting plasma insulin (mU/L)	6.23(4.48,8.67)	9.15(6.1,13.71)
IN30 (mU/L)	52.44(36.13,77.27)	26.61(15.23,45.33)
D30	12.83(8.18,20.7)	3.53(1.81,6.28)
IN2H (mU/L)	32.37(20.51,48.21)	51.12(30.67,84.97)
G_{AUC}	15.33(14.07,16.51)	24.53(21.99,28.57)
I_{AUC}	79.74(57.4,114.97)	69.58(42.48,111.38)
STU1	459.1(336.75,586.29)	127.66(46.83,217.38)
STU2	154.01(126.01,185.68)	67.87(25.59,125.04)
Gutt-ISI	79.85(71.52,88.27)	50.34(38.62,62.42)

WC, waist circumference; WHR, waist-to-hip ratio; HbA1c, glycated hemoglobin A1c; SBP, systolic blood pressure; DBP, diastolic blood pressure; PG30, 30 min plasma glucose, PG2H, 2 h plasma glucose, IN30, 30 min plasma insulin, IN2H, 2 h plasma insulin, GAUC, area under the curve of the glucose from 0 to 120 min; IAUC, area under the curve of the insulin from 0 to 120 min; D30, change of insulin levels/change of glucose levels from 0 to 30 min; STU1, Stumvoll first phase insulin secretion; STU2, Stumvoll second phase insulin secretion. Data are presented as mean+SD, median(interquartile range), or N (%).

Supplementary Table S2. Linkage disequilibrium of three SNPs.

		**rs112377266**	**rs61002819**	**rs536028004**
rs112377266	r^2	1	0.001	0.011
	D'	1	0.054	0.733
rs61002819	r^2	0.001	1	0.1
	D'	0.054	1	0.995
rs536028004	r^2	0.011	0.1	1
	D'	0.733	0.995	1

The pairwise linkage disequilibrium data of three SNPs are demonstrated. D' and r^2 values calculated based on our genotype data.

Supplementary Table S3. Antibodies of immunoblots and immunostainings.

Antibodies	Source	Identifier
Rabbit monoclonal anti-BRSK2	Cell Signaling	Cat#5460; RRID:AB_10838403
Rabbit polyclonal anti-PDX1	Cell Signaling	Cat#5679; RRID:AB_10706174
Rabbit polyclonal anti-MAFA	Santa Cruz	Cat#sc-66958; RRID:AB_2234386
GAPDH (1A6) monoclonal antibody-HRP	Bioworld	Cat#MH001H; RRID:AB_2857326
Mouse monoclonal anti-α-tubulin	Sigma-Aldrich	Cat#T4026; RRID:AB_477577
Rabbit polyclonal anti-β-actin	Bioworld	Cat#AP0731; RRID:AB_2797410
Mouse monoclonal anti-insulin	Servicebio	Cat#GB13121; RRID:AB_2811186
Goat polyclonal anti-insulin	Santa Cruz	Cat#sc-7839; RRID:AB_2800506
Rabbit polyclonal anti-glucagon	Servicebio	Cat#GB11097; RRID:AB_2811187
Rat monoclonal anti-somatostatin	Abcam	Cat#ab30788; RRID:AB_778010
Rabbit polyclonal anti-pancreatic polypeptide	Phoenlx Pharmaceuticals	Cat#H-054-02; RRID:N/A
Mouse monoclonal Syntaxin 1A (STX1A)	Proteintech	Cat#66437-1-Ig; RRID:AB_2881807
Rabbit polyclonal SNAP25	Proteintech	Cat#14903-1-AP; RRID:AB_2192051
Rabbit polyclonal VAMP2	Proteintech	Cat#10135-1-AP; RRID:AB_2256918
Rabbit anti-phospho-AMPK substrate motif [LXRXX(pS/pT)	Cell Signaling	Cat#5759; RRID: AB_10949320
Rabbit monoclonal anti-phospho-Akt (Thr308)	Cell Signaling	Cat#13038; RRID: AB_2629447
Rabbit monoclonal anti-phospho-Akt (Ser473)	Cell Signaling	Cat#4060; RRID: AB_2315049
Anti-mouse IgG	Cell Signaling	Cat#7076; RRID: AB_330924
Anti-rabbit IgG	Cell Signaling	Cat#7074; RRID: AB_2099233
Donkey anti-rabbit 488	Proteintech	Cat#A21206; RRID: AB_2535792
Donkey anti-Mouse 594	Invitrogen	Cat#A32744; RRID: AB_2762826
Donkey anti-Goat 488	Yeasen Biotech	Cat#34306ES60; RRID: N/A
Donkey anti-Rat 594	Yeasen Biotech	Cat#34412ES60; RRID: N/A

Supplementary Table S4. Primer sequences of qRT-PCR.

Gene symbol	Forward (5'~3')	Reverse (5'~3')	PCR product (bp)
**qPCR primers (mouse)**			
Actb	GGCTGTATTCCCCTCCATCG	CCAGTTGGTAACAATGCCATGT	154
Arpppo	GAAACTGCTGCCTCACATCCG	GCTGGCACAGTGACCTCACACG	147
Brsk2	ACCTGCTGCTAGATGAGAGGA	CTCGCCCCGAATCACTTCC	134
Scd1	TTCTTGCGATACACTCTGGTGC	CGGGATTGAATGTTCTTGTCGT	98
Pparγ	TGCAGCCTCAGCCAAGTTGAA	TTCCCGAACTTGACCAGCCA	77
Mcp1	CACTCACCTGCTGCTACTCA	GCTTGGTGACAAAAACTACAGC	117
F4/80	TCATCAGCCATGTGGGTACAG	CACAGCAGGAAGGTGGCTATG	70
Il6	TAGTCCTTCCTACCCCAATTTCC	TTGGTCCTTAGCCACTCCTTC	76
Fasn	GGAGGTGGTGATAGCCGGTAT	GGAGGTGGTGATAGCCGGTAT	140
Dgat1	TCCGTCCAGGGTGGTAGTG	TGAACAAAGAATCTTGCAGACGA	199
Dgat2	GCGCTACTTCCGAGACTACTT	GGGCCTTATGCCAGGAAACT	172
Srebp1c	TGACCCGGCTATTCCGTGA	CTGGGCTGAGCAATACAGTTC	61
Pepck	CTGCATAACGGTCTGGACTTC	CAGCAACTGCCCGTACTCC	159
Pc1	TGGGTTCCTCTCAGAGCGAG	GTCTCCCATCTTGCGGACC	100
G6pc1	CGACTCGCTATCTCCAAGTGA	GTTGAACCAGTCTCCGACCA	173
Fbp1	AGTCGTCCTACGCTACCTGTG	GGGGATCGAAACAGACAACAT	102
**qPCR primers (human)**			
ACTB	CATGTACGTTGCTATCCAGGC	CTCCTTAATGTCACGCACGAT	250
BRSK2	ACATCCGCATCGCAGACTTT	CGCAAGTTGTCATCGTCGAAG	205
